# The Metabolic Building Blocks of a Minimal Cell

**DOI:** 10.3390/biology10010005

**Published:** 2020-12-24

**Authors:** Mariana Reyes-Prieto, Rosario Gil, Mercè Llabrés, Pere Palmer-Rodríguez, Andrés Moya

**Affiliations:** 1 Evolutionary Systems Biology of Symbionts, Institute for Integrative Systems Biology, University of Valencia and Spanish Research Council, Paterna, 46980 Valencia, Spain; reyes_ber@gva.es (M.R.-P.); rosario.gil@uv.es (R.G.); 2 Sequencing and Bioinformatics Service, Foundation for the Promotion of Sanitary and Biomedical Research of the Valencia Region, 46020 Valencia, Spain; 3 Department of Mathematics and Computer Science, University of Balearic Islands, 07122 Palma de Mallorca, Spain; merce.llabres@uib.es (M.L.); pere.palmer@uib.es (P.P.-R.); 4 Genomic and Health Area, Foundation for the Promotion of Sanitary and Biomedical Research of the Valencia Region, 46020 Valencia, Spain; 5 Centro de Investigación Biomédica en Red en Epidemiología y Salud Pública, 28029 Madrid, Spain

**Keywords:** metabolic networks, minimal gene set machinery, directed acyclic graphs, minimal cells

## Abstract

**Simple Summary:**

Manufacturing artificial living cells would open endless research possibilities in basic and applied sciences. With this motivation, many research groups are developing methodologies to construct a stable minimal cell that is capable of achieving metabolic homeostasis, reproducing, and evolving in a controlled environment. Using as a template the gene set for a minimal cell proposed previously by Gil and coworkers, we have put together a network depicting its inferred minimal metabolism needed for life. This network has been further compressed as a metabolic Directed Acyclic Graph (m-DAG) in order to better visualize its topology and to find its essential reactions (i.e., critical reactions to maintain the network’s connectivity). We have also compared this minimal m-DAG to those of the smallest natural genome known until now and a synthetic minimal cell created in the laboratory. The modeling of m-DAGs based on minimal metabolisms can be a first approach for the synthesis and manipulation of minimal cells.

**Abstract:**

Defining the essential gene components for a system to be considered alive is a crucial step toward the synthesis of artificial life. Fifteen years ago, Gil and coworkers proposed the core of a putative minimal bacterial genome, which would provide the capability to achieve metabolic homeostasis, reproduce, and evolve to a bacterium in an ideally controlled environment. They also proposed a simplified metabolic chart capable of providing energy and basic components for a minimal living cell. For this work, we have identified the components of the minimal metabolic network based on the aforementioned studies, associated them to the KEGG database and, by applying the MetaDAG methodology, determined its Metabolic Building Blocks (MBB) and reconstructed its metabolic Directed Acyclic Graph (m-DAG). The reaction graph of this metabolic network consists of 80 compounds and 98 reactions, while its m-DAG has 36 MBBs. Additionally, we identified 12 essential reactions in the m-DAG that are critical for maintaining the connectivity of this network. In a similar manner, we reconstructed the m-DAG of JCVI-syn3.0, which is an artificially designed and manufactured viable cell whose genome arose by minimizing the one from *Mycoplasma mycoides* JCVI-syn1.0, and of “*Candidatus* Nasuia deltocephalinicola”, the bacteria with the smallest natural genome known to date. The comparison of the m-DAGs derived from a theoretical, an artificial, and a natural genome denote slightly different lifestyles, with a consistent core metabolism. The MetaDAG methodology we employ uses homogeneous descriptors and identifiers from the KEGG database, so that comparisons between bacterial strains are not only easy but also suitable for many research fields. The modeling of m-DAGs based on minimal metabolisms can be the first step for the synthesis and manipulation of minimal cells.

## 1. Introduction

One of the most ambitious aspirations of modern biology is to synthesize artificial living cells. Manufacturing a cell opens endless research possibilities, both in basic and advanced sciences, and it would be critical and a turning point in fields from medicine to evolutionary biology. To reduce the levels of difficulty on this task, most efforts are focused on the synthesis of minimal cells. On the one hand, they will help by increasing our understanding of living systems; on the other hand, they can be used as capsules for the introduction of genetic material to customize cells for applied purposes [1]. Several complementary paths have been followed in search of the proper technology and methods to design this fabricated cell. The most commonly used are the bottom–up and top–down approaches [2,3,4].

The bottom–up approach consists of the assembly, piece by piece, of each non-living biological component (i.e., a self-replicating nucleic acid, a metabolic machinery, and an encapsulating structure; [5]) in order to get a system that could be considered alive. The resulting products of this approach are called “protocells” [6,7]. No comparable system has been successfully constructed yet, but there have been developments on this front, with the designing of more refined cell-like compartments [8].

The top–down approach consists of deconstructing living cells [4,9]. Taking modern cells with reduced genomes as a starting point, it aims at further simplifying them by removing dispensable genetic material. Experimental (genome-wide analyses by massive transposon mutagenesis, antisense RNA, and systematic gene knockout) and computational approaches (including comparative genomics, comparative proteomics and in silico cell modeling) have been used to characterize a set of essential and sufficient genes to compose a living cell, that is, the core of a minimal bacterial genome [10]. Experimentally, genes are considered to be essential based on indirect evidence from systematic and genome-wide inactivation or the inhibition of each individual gene present in a genome (compiled in http://www.essentialgene.org/ [11]). Comparative genomics has also been broadly used, assuming that genes that are common between distant organisms are prone to be essential [12]. In addition, naturally reduced genomes from bacteria with a host-associated lifestyle have been used for comparisons regarding gene content, because they must be approaching a minimal genome [13,14]. The merging of these studies demonstrated the relevance of considering that essential functions can be performed by alternative and unrelated (non-orthologous) gene products. Comparative studies only retrieve genes involved in functions for which there is no alternative in nature (e.g., the complex translational machinery), while a minimal genome must also include all genes essential to maintain metabolic homeostasis [15].

There is a third approach for the construction of a minimal genome that searches for the biochemical and modular description of well-defined pathways needed to perform all essential functions [16]. Despite some major challenges needing to be addressed, this approach allows a function-by-function debugging to reach self-replication, and it suggests a good starting point for the ultimate synthesizing of a minimal genome able to sustain an artificial minimal cell. The potentiality of chemically synthesizing genomic segments or complete genomes and confining them into pre-existing cells has revolutionized the study of minimal cells [17]. The design of a truly minimal genome and its metabolic network can also benefit from computational whole-genome sequence rewriting and a design-build-test in silico approach, preceding the chemical synthesis of a customized genome [18].

A cohesive metabolic network proposal can lead the path to the synthesis of minimal cells. A minimal cell would depend on a minimal set of anabolic pathways to convert and assemble its biomolecule building blocks with the use of the energy and nutrients available in the environment, to reach metabolic homeostasis, and to achieve cellular growth and reproduction. Nevertheless, there is scientific consensus regarding the existence of a variety of minimal metabolic schemes that are ecologically dependent and able to sustain a universal genetic machinery [19]. The simplest cell should be chemoorganoheterotrophic (i.e., an organism using organic compounds as carbon and energy sources), living in a nutrient-rich medium, in which the major metabolites (glucose, fatty acids, nitrogenous bases, amino acids, and vitamins) must be available without limitation, since this cell would not be able to synthesize them. Nevertheless, considering the adaptability of bacterial heterotrophic metabolisms, different metabolic schemes can be envisaged. The metabolic chart proposed by Gil and coworkers in 2004 [15] using a top–down approach, by performing a comprehensive analysis of all previous computational and experimental attempts to define a minimal genome, was based on the metabolic functions that were preserved in highly reduced genomes completely sequenced at that time, from endosymbiotic mutualistic or parasitic bacteria. The proposed core of the minimal genome encoded the costless pathways that would allow the cell to perform the selected metabolic functions. In order to maintain a coherent metabolic functionality, some pathways that were not present in some of the reduced genomes used in the aforementioned study were also incorporated, because their lack reflected a high dependence of their hosts. Likewise, the group of Craig Venter also explored this area and presented their list of essential genes for a minimal bacterium in 2006 [20]. Both sets of genes and the coherence of this metabolic network were further explored by Gabaldón et al. (2007) [19].

Metabolic networks determine the physiology and biochemistry of a cell. They are made of three components: the metabolic pathways, the chemical reactions involved in the metabolism, and the regulatory interactions of these reactions. Metabolic networks tend to be highly complex, even for simple organisms. For example, if we consider the metabolism of porphyrin and chlorophyll which is present in some animals, plants, fungi, bacteria, and archaea, we get a metabolic pathway map of 135 nodes and 181 edges in the reference pathway in the KEGG database (pathway: map00860). A pathway map with so many components is very difficult to visualize, especially when we are interested in the pathway topology. To this extent, it is highly advantageous to suitably reduce the number of nodes in order to visualize the network more precisely. Alberich and coworkers (2017) designed a methodology called MetaDAG [21], which consists of the contraction into a single node of those reactions that are strongly connected in the genome-wide reaction graphs. In this way, the resulting graph is a Directed Acyclic Graph (DAG), called a metabolic DAG (m-DAG), that preserves the network topology (i.e., the original relations between reactions) while it allows easy human exploration and visualization. One advantage of directed acyclic graphs is that they do not have cycles repeatedly producing and consuming the same metabolite. This methodology also creates reaction graphs and m-DAGs from multiple genomes, which can be used to calculate the core- and pan-metabolisms of a group of bacteria of interest as well as compare genomes by their m-DAGs in a novel manner. The MetaDAG methodology can also be of importance for large in silico analyses. By compressing metabolic networks and making them “simpler”, algorithms and computer analyses could also be less time consuming. Just as important, less computational resources would be needed, making it easier for researchers to work with a large number of genome wide m-DAGs, bacterial consortia m-DAGs, multiple symbiosis analyses, or even environmental metabolomics.

For the current work, we constructed the minimal metabolic network from the theoretical minimal gene set machinery revised in Gabaldón and coworkers (2007) [19], and compared it to the smallest genome of a live organism known to date [22], and to the genome of a semisynthetic bacteria produced by Craig Venter’s group in 2016 [17]. Despite the great efforts being done to homogenize gene and enzyme names in databases, due to how they have been discovered and described throughout history, some of their names are still associated with taxonomically related organisms. For this reason, to avoid any remaining biases toward any group of organisms and any need for synonym lists, we propose a minimal metabolic network defined by reactions and compounds instead of genes. Moreover, another of the advantages of our methodology is that it is essentially universal, since it uses homogenous identifiers and descriptors, so that researchers can easily associate the involved reactions and compounds to genes of bacterial genomes with different phylogenetic backgrounds, even to synthetic genomes as proven in this study. Finally, it can also be applied to bacterial consortia in order to detect the metabolic interactions between partners and communities.

## 2. Materials and Methods

### 2.1. Inference of Minimal Metabolic Networks

The metabolic networks for this study were inferred from the reviewed version of the theoretical minimal genome described by Gabaldón et al. (2007) [19], the genome of “*Ca.* Nasuia deltocephalinicola” str. NAS-ALF [22] (which is also publicly available in the new version of the SymGenDB [23]), and the genome of JCVI-syn3.0, which is an artificial viable cell created by Hutchison and coworkers [17]. We first searched for all protein-coding genes in each genome for which an enzymatic activity has been assigned and then searched for the corresponding reactions in KEGG. 

### 2.2. Reconstruction of the Directed Acyclic Graph of Metabolic Networks 

Using the above obtained information, which is a set of reactions for each metabolic network, we generated the corresponding reaction graph that models the relationship between reactions in terms of shared metabolites. A reaction graph, denoted by *RG* = (*R*, *E_R_*), is a directed graph with a set of nodes *R* that are reactions and whose edges are defined as follows: there is an edge pointing from reaction *R_i_* to reaction *R_j_* if, and only if, a metabolite produced by reaction *R_i_* is a substrate in reaction *R_j_*. The fact that it is a directed graph establishes a natural production/consumption order between two reactions—that is, what is produced by *R_i_* is then consumed by *R_j_*. Before generating the directed graph, we manually curated it to remove redundancies (enzymes encoded by orthologous genes).

In order to analyze the reaction graph in a visually friendly manner, we used the MetaDAG methodology [21]. In a reaction graph, two reactions *R_i_*, *R_j_* are said to be biconnected if there is a path in each direction between them. A strongly connected component of a reaction graph is a subgraph such that every pair of reactions in it are biconnected. These strongly connected components are contracted in a single node. The reactions that are not biconnected to any other reaction become a node by themselves. Each node is called a Metabolic Building Block (MBB for short), and the MetaDAG software automatically assigns an ID to each MBB. When each MMB is contracted to a single vertex, the resulting quotient graph is a metabolic Directed Acyclic Graph (m-DAG for short). Thus, the m-DAG is defined as follows: its nodes are the MBBs obtained from the reaction graph, and there is an edge between two MBBs, MBB_1_ and MBB_2_, if there is an edge in the reaction graph from a reaction in MBB_1_ to a reaction in MBB_2_. We denote by *Gm* the m-DAG, thus *Gm* = (*N*, *E*) where *N* is the set of MBBs and *E* is the edges between them such that

(MBB_1_, MBB_2_) ∈ *E* ⇔ ∃ *R_i_* ∈ MBB_1_ ∧ ∃ *R_j_* ∈ MBB_2_ | (*R_i_*, *R_j_*) ∈ *E_R_*

MBBs contracting only one reaction and whose removal disconnects the reaction graph are considered essential reactions because they are crucial to maintain the network’s connectivity.

## 3. Results and Discussion

### 3.1. Theoretical Minimal Metabolic Network

**Figure 1:**
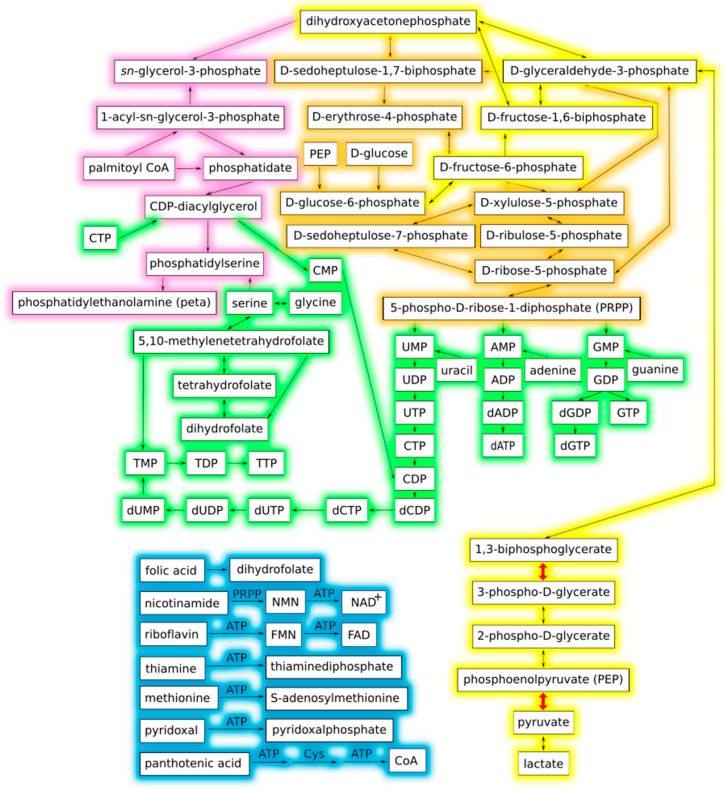
Interaction graph of the proposed theoretical minimal metabolic network adapted from Gabaldón et al. (2007) [19]. Line colors denote metabolic categories: yellow, glycolysis; orange, pentose phosphate pathway; pink, phospholipid metabolism; green, nucleotide metabolism; blue, coenzyme metabolism. The two glycolytic steps in which ATP is produced by substrate-level phosphorylation are depicted with thicker red arrows, and correspond to reactions R01512 and R00200 in Table 1. The reaction graph of this same network is presented in Figure 2 for comparison.

The first step toward the creation of the minimal metabolic network was to extrapolate the list of genes and enzymes belonging to the set presented by Gabaldón and coworkers (2007) [19] (Figure 1 and Table S1) to obtain KEGG reaction identifiers (IDs). We used the complete reaction, compound, and enzyme database from KEGG and created the reaction graph by joining the reactions where metabolites were shared (see Section 2.2 for the complete explanation). The idea behind using the complete KEGG catalog is to avoid biases toward a specific phylogenetic group of bacteria.

**Figure 2:**
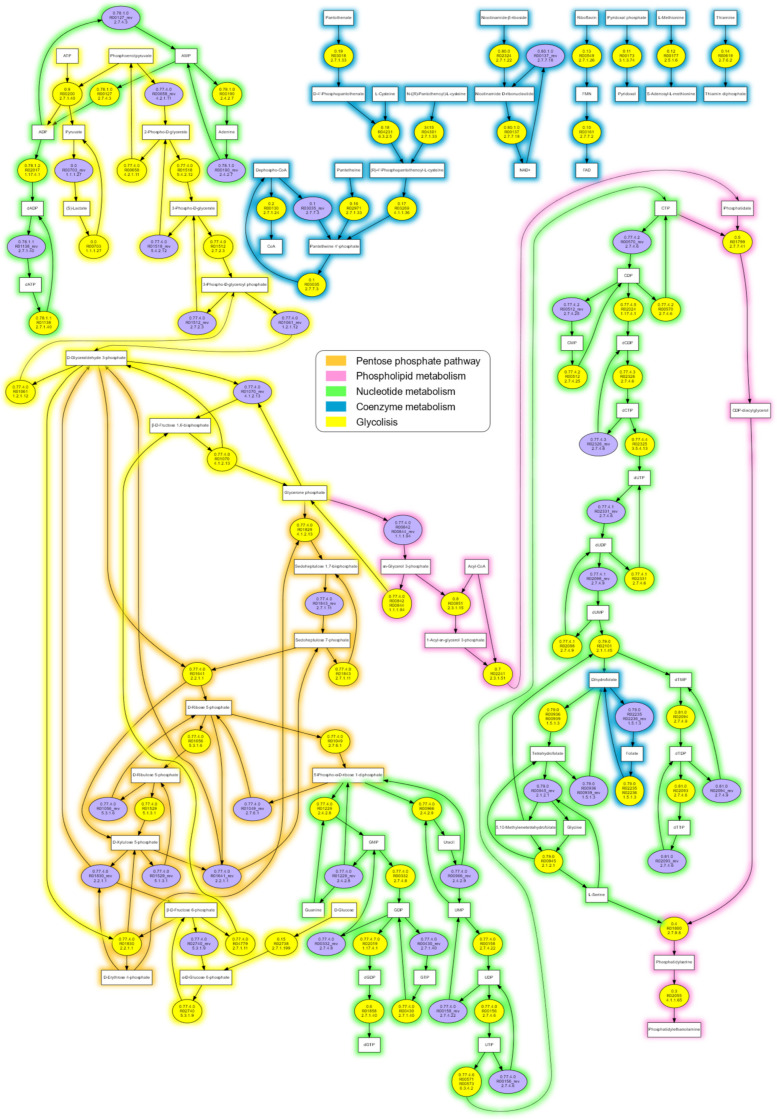
The reaction graph of the proposed theoretical minimal metabolic network represented in Figure 1, obtained using data from the KEGG database. The yellow filled circles are the reactions with their KEGG ID and E.C. numbers, and the purple filled circles are the reverse reaction of the yellow filled circles, when appropriate. Line colors denote metabolic categories. A full-size representation can be seen as Figure S1.

This methodology gave us a resulting reaction graph with some redundancies (i.e., different enzymes encoded by orthologous genes participating in the same metabolic pathways), so we manually curated this graph to include only one copy of each reaction and their corresponding metabolites needed for a functional cell. The reaction graph obtained is composed of 98 reactions and 80 metabolites (Figure 2). The fact that our model replicates almost entirely the figure of Gabaldón et al. (2007) [19] (Figure 1), validates our methodology. Table 1 presents the complete list of reactions, substrate, and product compounds as well as their KEGG identifiers used to reconstruct the minimal metabolic network.

### 3.2. The MetaDAG Methodology: Analysis of the Composition and Connectivity of a Network at a Glance 

Despite the fact that the reaction graph of the theoretical minimal organism constructed in this work has only 98 reactions and 80 metabolites, it is difficult to visualize the detailed relationships between the reactions that make up the network’s connectivity (Figure 2). To solve this problem, we used the MetaDAG methodology [21] to generate an m-DAG of the manually curated reaction graph. An m-DAG is a suitable reduction of a metabolic network. Namely, the reactions that are connected by multiple paths, which are the strongly connected components of the metabolic network, are contracted into one single MBB, which can be considered a robust subgraph in the reaction graph. Moreover, those MBBs that only represent a reaction that is not biconnected to any other reaction are essential to maintain the network connectivity. In this sense, the m-DAG provides a modularity of the reaction graph that keeps the information of robustness and connectivity of the metabolic network. 

**Figure 3:**
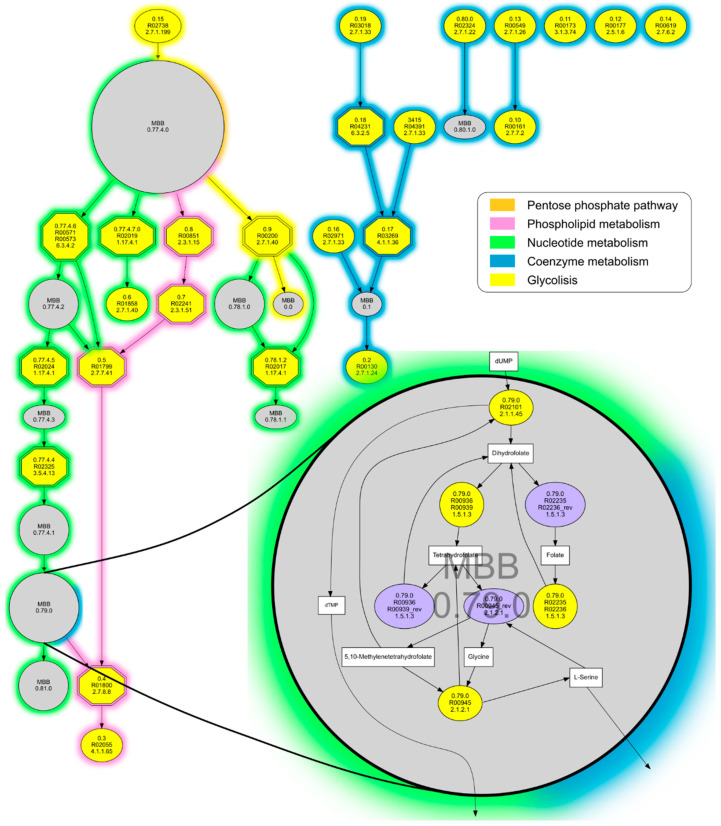
m-DAG of the metabolism of a theoretical minimal bacterial cell. Single reactions appear in yellow, contracted MBBs in grey, and the essential reactions as hexagons with double lines. Line colors denote metabolic categories. MBB 0.79.0 is zoomed in as an example of how a strongly connected component, which is a cyclic subgraph formed by 7 reactions and 7 compounds, is reduced to one node in our m-DAG.

The m-DAG we obtained from the minimal metabolic reaction graph (Table 1, Figure 3) has a total of 36 nodes, 25 of them corresponding to single reactions (yellow nodes) and 11 to contracted MBBs (gray nodes). Clearly, there are seven connected components in this network, the biggest one covering the central metabolism of the hypothetical minimal organism, while the rest are the reactions that synthesize the essential cofactors needed for the proper functionality of the complete cell. 

In addition, essential reactions (i.e., those whose removal reduces the network’s connectivity increasing the number of connected components) can be easily identified using this approach (hexagons with double lines in Figure 3). Table 2 is a list of the 12 essential reactions we found in the minimal metabolic network under study and the metabolic pathways where they participate. They are involved in purine and pyrimidine metabolism, glycerophospholipid metabolism, glycolysis and pantothenate, and CoA biosynthesis. Purines and pyrimidines are the most abundant metabolic substrates for all living organisms. They are essential components for the synthesis of DNA and RNA, and they also participate in the biosynthesis of energy nucleotides and are vital cofactors for cell survival and reproduction. Hence, purines and their by-products widely participate in biological processes. Glycerophospholipids are pivotal structural components of the cell membranes, but they are also precursors of many essential biological molecules and participate in cell signaling and other cellular processes [24]. Glycolysis is the first step in the breakdown of glucose to extract energy for cellular metabolism by creating high-energy molecules. It is considered an ancient metabolic pathway [25], and its prevalence in organisms is nearly ubiquitous.

We consider that what we call “essential reactions”, easily highlighted by the MetaDAG methodology, can be of crucial importance in many fields of research. Probably, the most logical and of vital importance is the idea that these reactions can help choose enzymes as potential drug targets, since the removal of these reactions breaks metabolic pathways, which can lead to the unviability of a cell. Considering that m-DAGs take into account complete genomes, and even complementary genomes (they can be calculated for two or more genomes together, to simulate complementary metabolic pathways within consortia), the resulting essential reactions are trustworthy in a sense that researchers might overlook an enzyme doing the same job as the one highlighted and, if they find it, it would be a new discovery not previously described for a specific metabolic pathway.

### 3.3. The m-DAG of “Candidatus Nasuia Deltocephalinicola”

In the case of a minimal metabolic network, each item included in the list of reactions and compounds is hypothetically essential for survival. When we extrapolate these results to living organisms possessing natural minimized genomes, such as pathogens or mutualist endosymbiotic bacteria, we should consider that their metabolism is a patchwork dependent on the host and, in many cases, also dependent on other bacteria with which they live in consortia. Therefore, the study of their networks’ connectivity has the potential of pointing out genes encoding critical steps that connect the different partners in a given pathway. Subsequently, the genes that encode those reactions can become targets for genetic engineering, and/or for mechanisms intended to regulate the cell metabolism; additionally, they might also have the potential to destroy the stability of the relationship, even killing the undesired organism in a parasitic relationship.

In order to compare the in silico minimal m-DAG with the m-DAG from a living organism with a naturally reduced genome, we constructed the m-DAG of “*Ca.* Nasuia deltocephalinicola” str. NAS-ALF (from now on referred to as *Nasuia* for simplicity; Supplementary Figure S2), one of the obligate endosymbiotic bacteria of the aster leafhopper *Macrosteles quadrilineatus* [22]. This endosymbiont possesses the smallest natural genome known so far, comprising 112,091 bp and only 138 protein-coding genes identified. The metabolic data needed to generate this m-DAG, including the complete list of its enzymes, reactions, and compoundswere also obtained from the KEGG database (Table S2). *Nasuia*’s m-DAG comprises 29 nodes included in 12 connected components, with 7 MBBs and 22 single reactions. Regarding the single reactions, five are essential (summarized in Table 3). 

It has been estimated that more than 60% of insects possess symbiotic bacteria inside their body tissues, and/or very often in a specialized cell type called bacteriocyte [26]. When these bacteria become endosymbionts, they lose their ability to interact with other organisms. Additionally, they become dependent on their respective hosts, and their genome is significantly reduced by the deletion of genes that become redundant or that are not needed in a rich environment such as the one they encounter within their hosts [15,27]. In addition, even though the niche is significantly rich for them, the insect host generally has a very incomplete diet by feeding on plant sap or seeds, or blood from mammals, so the bacteria become their helpers for the production of essential amino acids, fatty acids, or vitamins [28,29]. The essential reactions of *Nasuia*’s m-DAG reveal exactly that. This organism works as a factory of the vitamins and amino acids that *M. quadrilineatus* needs to survive. Moreover, this bacterium is part of a consortium with “*Candidatus* Sulcia muelleri” str. ALF [22]. It is widely accepted that the endosymbiotic relationship between insects and bacteria, dating from 10 to several hundred millions of years, allowed the proliferation of insects and their diversification in almost any ecological niche [30,31]. Obviously, if the reactions that link the metabolic routes disappear (either naturally or due to targeted modification of those genes), this association would be affected to the point of the possible death of the host. 

A direct comparison between the reactions and compounds that make up the in silico m-DAGs of the theoretical minimal cell and *Nasuia* would not be significant due to their dissimilar lifestyles. What we can easily assess is the topology of the networks. At first glance, it is striking that the smallest genome found in nature has fewer nodes than the in silico m-DAG. The dependence of this endosymbiotic bacteria to its host and to its second co-obligate endosymbiont explains this phenomenon.

### 3.4. The First Semisynthetic Viable Cell and Its m-DAG’s Reconstruction

**Figure 4:**
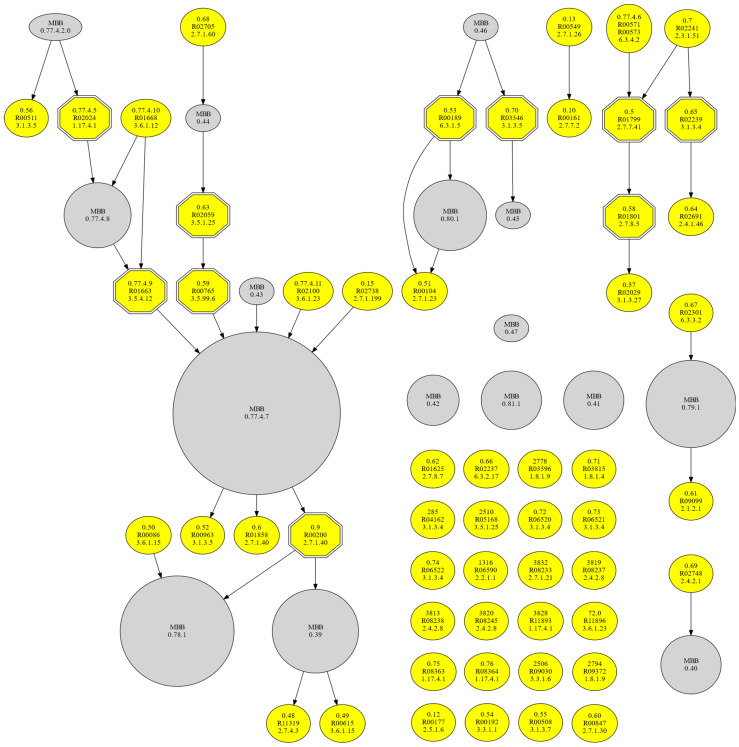
m-DAG of the metabolism of JCVI-syn3.0. Single reactions appear in yellow, contracted MBBs in grey, and the essential reactions as hexagons with double lines.

To complete our comparative analysis, we constructed the m-DAG of JCVI-syn3.0, which is an artificially designed and manufactured viable cell whose genome arose by minimizing the one from *Mycoplasma mycoides* JCVI-syn1.0 created by Hutchison et al. in 2016 [17]. To do so, we used the list of enzymes presented in their article and converted it into a list of reactions and compounds, compared them to our minimal metabolic network (Table S3), and created the reaction graph of JCVI-syn3.0 and its eventual m-DAG (Figure 4).

JCVI-syn3.0 m-DAG is formed by 34 connected components, with a total of 70 nodes, 54 of them corresponding to single reactions, and 16 contracted MBBs. Ten reactions are essential (summarized in Table 4), that is, indispensable to maintain the connectivity of the network.

Once again, the essential reactions are involved in the metabolism of nucleotides, phospholipids, and coenzymes, even though there are significant differences between the list of reactions included in the reconstruction of JCVI-syn3.0 and the metabolic minimal network (Supplementary Table S3). JCVI-syn3.0 has 155 reactions included in its reaction graph, while our minimal network reaction graph has only 63 (98 when taking reverse reactions into account). The explanation for these differences is that the minimal network defined by Gil and coworkers (2004) [15] considers the minimal bacterium to live in a controlled and nutrient-rich environment, while JCVI-syn3.0 includes some metabolic pathways that are essential for the specific necessities of *M. mycoides*, its reproduction, and its survival. Interestingly enough, two reactions are essential for both networks (R02024 and R00200), while others participate closely in the same pathways (e.g., R01800 and R01801), which may be useful information for genetic engineering purposes. 

### 3.5. Resemblance of the MBBs of the Minimal m-DAGs

In order to contrast the MBBs of the three m-DAGs constructed in this study, Table 5 shows the correspondence among them. The list of enzymes and the definition of each reaction is presented in Supplementary Table S4.

## 4. Conclusions

The construction of the minimal metabolic reaction graph and its consequent m-DAG presented in this work can be of great use in the field of synthetic biology. The composition of compounds and reactions that we present can easily be extrapolated to any phylogenetically diverse bacteria of interest considering that we did not focus specifically on genes. Chemistry and molecular biology technologies are also thriving. Thus, the in silico design of bacteria with the small number of metabolic genes described in this paper may be more feasible than previously thought.

## Figures and Tables

**Table 1:** Reactions, enzymes, and compounds of the minimal metabolic network presented in Figure 2. Reversible reactions are denoted by the superscript *r*. MBB IDs are the identification numbers of the metabolic building blocks to which each reaction is contracted into, according to the MetaDAG analysis (Figure 3).

Substrate KEGG ID	Reaction ID	Enzyme Name (E.C. Number)	Definition	Product KEGG ID	MBB ID
C00020	R00127 *^r^*	adenylate kinase (2.7.4.3)	ATP + AMP ↔ 2 ADP	C00008	0.15
C00882	R00130	dephospho-CoA kinase (2.7.1.24)	ATP + Dephospho-CoA → ADP + CoA	C00010	0.2
C00455	R00137 *^r^*	nicotinamide-nucleotide adenylyltransferase (2.7.7.1)	Diphosphate + NAD+ ↔ ATP + Nicotinamide d-ribonucleotide	C00003	0.80.1.0
C00015	R00156 *^r^*	nucleoside-diphosphate kinase (2.7.4.6)	ATP + UDP ↔ ADP + UTP	C00075	0.77.4.0
C00105	R00158 *^r^*	UMP/CMP kinase (2.7.4.14)	ATP + UMP ↔ ADP + UDP	C00015	0.77.4.0
C00061	R00161	FAD synthase (2.7.7.2)	ATP + FMN → Diphosphate + FAD	C00016	0.10
C00018	R00173	pyridoxal phosphatase (3.1.3.74)	Pyridoxal phosphate + H_2_O → Pyridoxal + Orthophosphate	C00250	0.11
C00073	R00177	methionine adenosyltransferase (2.5.1.6)	ATP + l-Methionine + H_2_O → Orthophosphate + Diphosphate + S-Adenosyl-l-methionine	C00019	0.12
C00020 + C0013	R00190 *^r^*	adenine phosphoribosyltransferase (2.4.2.7)	AMP + Diphosphate ↔ Adenine + 5-Phospho-alpha-d-ribose 1-diphosphate	C00147 + C00119	0.78.1.0
C00074 + C00008	R00200	pyruvate kinase (2.7.1.40)	ADP + Phosphoenolpyruvate → ATP + Pyruvate	C00022	0.9
C00144	R00332 *^r^*	guanylate kinase (2.7.4.8)	ATP + GMP ↔ ADP + GDP	C00035	0.77.4.0
C00044	R00430 *^r^*	pyruvate kinase (2.7.1.40)	GTP + Pyruvate ↔ GDP + Phosphoenolpyruvate	C00035	0.77.4.0
C00055	R00512 *^r^*	(d)CMP kinase (2.7.4.25)	ATP + CMP ↔ ADP + CDP	C00112	0.77.4.2
C00255	R00549	riboflavin kinase (2.7.1.26)	ATP + Riboflavin → ADP + FMN	C00061	0.13
C00112	R00570 *^r^*	nucleoside diphosphate kinase (2.7.4.6)	ATP + CDP ↔ ADP + CTP	C00063	0.77.4.2
C00075	R00571, R00573	CTP synthase (6.3.4.2)	ATP + UTP + Ammonia → ADP + Orthophosphate + CTP	C00063	0.77.4.6
C00378	R00619	thiamine diphosphokinase (2.7.6.2)	ATP + Thiamine → AMP + Thiamin diphosphate	C00068	0.14
C00631	R00658 *^r^*	enolase (4.2.1.11)	2-Phospho-d-glycerate ↔ Phosphoenolpyruvate + H_2_O	C00074	0.77.4.0
C00186	R00703 *^r^*	lactate dehydrogenase (1.1.1.27)	(S)-Lactate + NAD+ ↔ Pyruvate + NADH + H+	C00022	0.0
C00093	R00842|R00844 *^r^*	sn-glycerol-3-phosphate dehydrogenase (1.1.1.94)	sn-Glycerol 3-phosphate + NAD+ ↔ Glycerone phosphate + NADH + H+	C00111	0.77.4.0
C00093 + C00040	R00851	arylamine N-acetyltransferase (2.3.1.15)	sn-Glycerol 3-phosphate + Acyl-CoA → 1-Acyl-sn-glycerol 3-phosphate + CoA	C00681	0.8
C00415	R00936|R00939 *^r^*	dihydrofolate reductase (1.5.1.3)	Dihydrofolate + NADH + H+ ↔ Tetrahydrofolate + NAD +	C00101	0.79.0
C00037 + C00143	R00945 *^r^*	glycine hydroxymethyltransferase (2.1.2.1)	5,10-Methylenetetrahydrofolate + Glycine + H_2_O ↔ Tetrahydrofolate + l-Serine	C00065 + C00101	0.79.0
C00105	R00966 *^r^*	uracil phosphoribosyltransferase (2.4.2.9)	UMP + Diphosphate ↔ Uracil + 5-Phospho-alpha-d-ribose 1-diphosphate	C00106 + C00119	0.77.4.0
C00117	R01049 *^r^*	phosphoribosylpyrophosphate synthetase (2.7.6.1)	ATP + d-Ribose 5-phosphate ↔ AMP + 5-Phospho-alpha-d-ribose 1-diphosphate	C00119	0.77.4.0
C00117	R01056 *^r^*	ribose-5-phosphate isomerase (5.3.1.6)	d-Ribose 5-phosphate ↔ D-Ribulose 5-phosphate	C00199	0.77.4.0
C00118	R01061 *^r^*	glyceraldehyde-3-phosphate dehydrogenase (1.2.1.12)	d-Glyceraldehyde 3-phosphate + Orthophosphate + NAD+ ↔ 3-Phospho-d-glyceroyl phosphate + NADH + H+	C00236	0.77.4.0
C05378	R01070 *^r^*	fructose-1,6-bisphosphate aldolase (4.1.2.13)	beta-d-Fructose 1,6-bisphosphate ↔ Glycerone phosphate + d-Glyceraldehyde 3-phosphate	C00111 + C00118	0.77.4.0
C00131	R01138 *^r^*	pyruvate kinase (2.7.1.40)	dATP + Pyruvate ↔ dADP + Phosphoenolpyruvate	C00206	0.78.1.1
C00119 + C00242	R01229 *^r^*	hypoxanthine phosphoribosyltransferase (2.4.2.8)	Guanine + 5-Phospho-alpha-d-ribose 1-diphosphate ↔ GMP + Diphosphate	C00144	0.77.4.0
C00361	R01858	pyruvate kinase (2.7.1.40)	dGDP + Phosphoenolpyruvate → dGTP + Pyruvate	C00286	0.6
C00008	R02017	ribonucleoside diphosphate reductase (1.17.4.1)	Thioredoxin + ADP → dADP + Thioredoxin disulfide + H_2_O	C00206	0.78.1.2
C00035	R02019	ribonucleoside diphosphate reductase (1.17.4.1)	GDP + Thioredoxin → dGDP + Thioredoxin disulfide + H_2_O	C00361	0.77.4.7.0
C00112	R02024	ribonucleoside diphosphate reductase (1.17.4.1)	Thioredoxin + CDP → dCDP + Thioredoxin disulfide + H_2_O	C00705	0.77.4.5
C00197	R01512 *^r^*	phosphoglycerate kinase (2.7.2.3)	ATP + 3-Phospho-d-glycerate ↔ ADP + 3-Phospho-d-glyceroyl phosphate	C00236	0.77.4.0
C00631	R01518 *^r^*	phosphoglycerate mutase (2,3-diphosphoglycerate-independent) (5.4.2.12)	2-Phospho-d-glycerate ↔ 3-Phospho-d-glycerate	C00197	0.77.4.0
C00199	R01529 *^r^*	ribulose-phosphate 3-epimerase (5.1.3.1)	d-Ribulose 5-phosphate ↔ d-Xylulose 5-phosphate	C00231	0.77.4.0
C00118 + C05382	R01641 *^r^*	transketolase (2.2.1.1)	Sedoheptulose 7-phosphate + d-Glyceraldehyde 3-phosphate ↔ d-Ribose 5-phosphate + d-Xylulose 5-phosphate	C00117 + C00231	0.77.4.0
C00063 + C00416	R01799 *^r^*	phosphatidate cytidyltransferase (2.7.7.41)	CTP + Phosphatidate → Diphosphate + CDP-diacylglycerol	C00269	0.5
C00065 + C00269	R01800	phosphatidylserine synthase (2.7.8.8)	-diacylglycerol + L-Serine → CMP + Phosphatidylserine	C00055 + C02737	0.4
C00279 + C00111	R01829	fructose-1,6-bisphosphate aldolase (4.1.2.13)	Glycerone phosphate + d-Erythrose 4-phosphate → Sedoheptulose 1,7-bisphosphate	C00447	0.77.4.0
C00118 + C05345	R01830 *^r^*	transketolase (2.2.1.1)	beta-d-Fructose 6-phosphate + d-Glyceraldehyde 3-phosphate → d-Erythrose 4-phosphate + D-Xylulose 5-phosphate	C00231 + C00279	0.77.4.0
C00363	R02093 *^r^*	nucleoside diphosphate kinase (2.7.4.6)	ATP + dTDP ↔ ADP + dTTP	C00459	0.81.0
C00364	R02094 *^r^*	thymidine monophosphate kinase (2.7.4.9)	ATP + dTMP ↔ ADP + dTDP	C00363	0.81.0
C00365	R02098 *^r^*	thymidine monophosphate kinase (2.7.4.9)	ATP + dUMP ↔ ADP + dUDP	C01346	0.77.4.1
C00143 + C00365	R02101	thymidylate synthase (2.1.1.45)	dUMP + 5,10-Methylenetetrahydrofolate → Dihydrofolate + dTMP	C00364 + C00415	0.79.0
C00040 + C00681	R02241	1-acyl-sn-glycerol-3-phosphate acyltransferase (2.3.1.51)	1-Acyl-sn-glycerol 3-phosphate + Acyl-CoA → Phosphatidate + CoA	C00416	0.7
C00458	R02325	dCTP deaminase (3.5.4.13)	dCTP + H2O → dUTP + Ammonia	C00460	0.77.4.4
C00705	R02326 *^r^*	nucleoside diphosphate kinase (2.7.4.6)	ATP + dCDP ↔ ADP + dCTP	C00458	0.77.4.3
C01346	R02331 *^r^*	nucleoside diphosphate kinase (2.7.4.6)	ATP + dUDP ↔ ADP + dUTP	C00460	0.77.4.1
C02737	R02055	phosphatidylserine decarboxylase (4.1.1.65)	Phosphatidylserine → Phosphatidylethanolamine + CO_2_	C00350	0.3
C00504	R02235|R02236 *^r^*	dihydrofolate reductase (1.5.1.3)	Folate + NADH + H+ ↔ Dihydrofolate + NAD+	C00415	0.79.0
C03150	R02324	ribosylnicotinamide kinase (2.7.1.22)	ATP + Nicotinamide-beta-riboside → ADP + Nicotinamide D-ribonucleotide	C00455	0.80.0
C00031	R02738	protein-Npi-phosphohistidine---d-glucose phosphotransferase (2.7.1.199)	Protein N(pi)-phospho-L-histidine + D-Glucose → Protein histidine + alpha-d-Glucose 6-phosphate	C00668	0.15
C00668	R02740 *^r^*	glucose-6-phosphate isomerase (5.3.1.9)	alpha-d-Glucose 6-phosphate ↔ beta-d-Fructose 6-phosphate	C05345	0.77.4.0
C00831	R02971	pantetheine kinase (2.7.1.34)	ATP + Pantetheine → ADP + Pantetheine 4’-phosphate	C01134	0.16
C00864	R03018	pantothenate kinase (2.7.1.33)	ATP + Pantothenate → ADP + D-4’-Phosphopantothenate	C03492	0.19
C01134	R03035 *^r^*	pantetheine-phosphate adenylyltransferase (2.7.7.3)	ATP + Pantetheine 4’-phosphate → Diphosphate + Dephospho-CoA	C00882	0.1
C03492	R04231 *^r^*	phosphopantothenoylcysteine synthetase (6.3.2.5)	CTP + D-4’-Phosphopantothenate + l-Cysteine ↔ CMP + Diphosphate + (R)-4’-Phosphopantothenoyl-L-cysteine	C04352	0.18
C04079	R04391 *^r^*	pantothenate kinase (2.7.1.33)	ATP + N-((R)-Pantothenoyl)-L-cysteine ↔ ADP + (R)-4’-Phosphopantothenoyl-l-cysteine	C04352	3415
C05345	R04779 *^r^*	6-phosphofructokinase (2.7.1.11)	ATP + beta-d-Fructose 6-phosphate ↔ ADP + beta-d-Fructose 1,6-bisphosphate	C05378	0.77.4.0
C04352	R03269	phosphopantothenoylcysteine decarboxylase (4.1.1.36)	(R)-4’-Phosphopantothenoyl-L-cysteine → Pantetheine 4’-phosphate	C01134	0.17
C05382	R01843 *^r^*	6-phosphofructokinase (2.7.1.11)	ATP + Sedoheptulose 7-phosphate ↔ ADP + Sedoheptulose 1,7-bisphosphate	C00447	0.77.4.0

**Table 2:** Essential reactions of the m-DAG constructed from the theoretical minimal gene set machinery needed for life.

Reaction ID	Metabolic Pathway
R02019	Purine metabolism
R02017	
R00571/R00573	Pyrimidine metabolism
R02024	
R02325	
R00851	Glycerolipid metabolism, Glycerophospholipid metabolism
R02241	
R01799	Glycerophospholipid metabolism
R01800	
R00200	Glycolysis, part of the pyruvate metabolism
R04231	Pantothenate and CoA biosynthesis
R03269	

**Table 3:** Essential reactions of the m-DAG of “*Ca.* Nasuia deltocephalinicola” str. NAS-ALF.

Reaction ID	Metabolic Pathway
R09372	Selenocompound metabolism
R00443	Purine metabolism, Glycerophospholipid metabolism
R03012	Histidine metabolism
R01163	Histidine metabolism
R01288	Cysteine and methionine metabolism, Sulfur metabolism

**Table 4:** Essential reactions of the m-DAG of JCVI-syn3.0.

Reaction ID	Metabolic Pathway
R02024	Pyrimidine metabolism
R01663	
R02059	Amino sugar and nucleotide sugar metabolism
R00765	
R00200	Glycolysis, part of the pyruvate metabolism
R00189	Nicotinate and nicotinamide metabolism
R03346	
R01799	Glycerophospholipid metabolism
R01801	
R02239	

**Table 5:** Comparison of the MBBs of the three networks under study. Every row lists the reactions belonging to the corresponding MBB and the enzymes involved in those reactions. The list includes only MBBs composed of at least three reactions (reverse included) or with fewer reactions but that are shared by at least two of the networks under study.

Model Cell	MBB ID	# Reactions	Reaction ID
**Minimal cell**	0.77.4.0	21	R00156 (2.7.4.6) ^r^, R00158 (2.7.4.22) ^r^, R00332 (2.7.4.8) ^r^, R00430 (2.7.1.40) ^r^, R00658 (4.2.1.11) ^r^, R00842|R00844 (1.1.1.94) ^r^, R00966 (2.4.2.9) ^r^, R01049 (2.7.6.1) ^r^, R01056 (5.3.1.6) ^r^, R01061 (1.2.1.12) ^r^, R01070 (4.1.2.13) ^r^, R01229 (2.4.2.8) ^r^, R01512 (2.7.2.3) ^r^, R01518 (5.4.2.12) ^r^, R01529 (5.1.3.1) ^r^, R01641 (2.2.1.1) ^r^, R01829 (4.1.2.13), R01830 (2.2.1.1) ^r^, R01843 (2.7.1.11) ^r^, R02740 (5.3.1.9) ^r^, R04779 (2.7.1.11)
	0.77.4.1	2	R02098 (2.7.4.9) ^r^, R02331 (2.7.4.6) ^r^
	0.77.4.2	2	R00512 (2.7.4.25) ^r^, R00570 (2.7.4.6) ^r^
	0.77.4.7.0	1	R02019 (1.17.4.1)
	0.78.1.0	2	R00127 (2.7.4.3) ^r^, R00190 (2.4.2.7) ^r^
	0.78.1.1	1	R01138 (2.7.1.40) ^r^
	0.78.1.2	1	R02017 (1.17.4.1)
	0.79.0	4	R00936|R00939 (1.5.1.3) ^r^, R00945 (2.1.2.1) ^r^, R02101 (2.1.1.45), R02235|R02236 (1.5.1.3) ^r^
	0.80.1.0	1	R00137 (2.7.7.18) ^r^
	0.81.0	2	R02093 (2.7.4.6) ^r^, R02094 (2.7.4.9) ^r^
**JCVI-syn 3.0**	0.39	6	R00014 (1.2.4.1), R00230 (2.3.1.8) ^r^, R00315 (2.7.2.1) ^r^, R02569 (2.3.1.12) ^r^, R03270 (1.2.4.1), R07618 (1.8.1.4) ^r^
	0.40	3	R01126 (3.1.3.5), R01132 (2.4.2.8) ^r^, R01863 (2.4.2.1) ^r^
	0.41	3	R02142 (2.4.2.8) ^r^, R02297 (2.4.2.1) ^r^, R02719 (3.1.3.5)
	0.42	2	R00921 (2.3.1.8) ^r^, R01353 (2.7.2.1) ^r^
	0.77.4.2.0	1	R00512 (2.7.4.25) ^r^
	0.77.4.7	49	R00158 (2.7.4.22) ^r^, R00289 (2.7.7.9) ^r^, R00291 (5.1.3.2) ^r^, R00332 (2.7.4.8) ^r^, R00430 (2.7.1.40) ^r^, R00505 (5.4.99.9) ^r^, R00658 (4.2.1.11) ^r^, R00959 (5.4.2.5) ^r^, R00966 (2.4.2.9) ^r^, R01015 (5.3.1.1) ^r^, R01049 (2.7.6.1)^r^, R01056 (5.3.1.6) ^r^, R01057 (5.4.2.7) ^r^, R01058 (1.2.1.9), R01061 (1.2.1.12) ^r^, R01066 (4.1.2.4) ^r^, R01067 (2.2.1.1), R01068 (4.1.2.13) ^r^, R01070 (4.1.2.13) ^r^, R01227 (3.1.3.5), R01229 (2.4.2.7)^r^, R01229 (2.4.2.8) ^r^, R01512 (2.7.2.3) ^r^, R01518 (5.4.2.12) ^r^, R01529 (5.1.3.1) ^r^, R01641 (2.2.1.1) ^r^, R01819 (5.3.1.8) ^r^, R01827 (2.2.1.2) ^r^, R01829 (4.1.2.13), R01830 (2.2.1.1) ^r^, R01843 (2.7.1.11) ^r^, R01967 (2.7.1.113) ^r^, R01968 (3.1.3.5)^r^, R01969 (2.4.2.1) ^r^, R02018 (1.17.4.1), R02019 (1.17.4.1), R02090 (2.7.4.8) ^r^, R02098 (2.7.4.9) ^r^, R02099 (2.7.1.21), R02102 (3.1.3.5), R02102 (3.1.3.89), R02147 (2.4.2.1)^r^, R02484 (2.4.2.1), R02568 (4.1.2.13) ^r^, R02739 (5.3.1.9) ^r^, R02740 (5.3.1.9) ^r^, R02749 (5.4.2.7) ^r^, R03321 (5.3.1.9) ^r^, R04779 (2.7.1.11)
	0.77.4.8	5	R01664 (3.1.3.5), R01664 (3.1.3.89), R01665 (2.7.4.25) ^r^, R01666 (2.7.1.74), R01667 (3.6.1.12)
	0.78.1	12	R00127 (2.7.4.3) ^r^, R00183 (3.1.3.5), R00185 (2.7.1.74) ^r^, R00190 (2.4.2.7) ^r^, R01138 (2.7.1.40) ^r^, R01547 (2.7.4.11) ^r^, R01547 (2.7.4.3) ^r^, R01561 (2.4.2.1) ^r^, R02017 (1.17.4.1), R02088 (3.1.3.5) ^r^, R02089 (2.7.1.76), R02557 (2.4.2.1) ^r^
	0.79.1	6	R00942 (6.3.2.17) ^r^, R00945(2.1.2.1) ^r^, R01220(1.5.1.5) ^r^, R01655 (3.5.4.9) ^r^, R03940 (2.1.2.9), R04241 (6.3.2.17) ^r^
	0.80.1	4	R00137 (2.7.7.18) ^r^, R01271 (2.4.2.12) ^r^, R02294 (2.4.2.1) ^r^, R02323 (3.1.3.5)
	0.81.1	4	R01567 (2.7.1.21), R01569 (3.1.3.5), R01569 (3.1.3.89), R02094 (2.7.4.9) ^r^
*Nasuia*	0.77.0	2	R00435 (2.7.7.6) ^r^, R00441 (2.7.7.6) ^r^
	0.78.0	2	R00375 (2.7.7.7) ^r^, R00376 (2.7.7.7) ^r^

Reversible reactions are denoted by the superscript r. Reactions depicted in blue are shared by the minimal and the synthetic (JCVI-syn3.0) metabolic networks. #: number of.

## Data Availability

All data is contained within this article or its own supplementary material.

## Supplementary Materials

The following are available online at https://www.mdpi.com/2079-7737/10/1/5/s1, Figure S1: Full size representation of the reaction graph of the proposed theoretical minimal metabolic network represented in Figure 2; Figure S2: The m-DAG of “*Ca.* Nasuia deltocephalinicola” str. NAS-ALF; Table S1: List of enzymes and reactions modified from Gabaldón et al. (2007) [19]; Table S2: Reactions and compounds that make up the m-DAG of “*Ca.* Nasuia deltocephalinicola” str. NAS-ALF; Table S3: Reactions included in the reconstruction of the JCVI-syn3.0 reaction graph and the minimal organism constructed for this work and the pathways in which each reaction (can) participates. Table S4: Names of the enzymes and definition of each reaction involved in the comparison of the MBBs of the three networks under study.
