# Supplementary material for: The Metabolic Building Blocks of a Minimal Cell
Source: Biology (Basel). 2020 Dec 24;10(1):5. doi: 10.3390/biology10010005 (PMC7824019; doi:10.3390/biology10010005)

The metabolic building blocks of a minimal cell

Mariana Reyes-Prieto, Rosario Gil, Mercè Llabrés, Pere Palmer and Andrés Moya

Supplementary material.

Table S1. List of enzymes and reactions modified from Gabaldon et. al. (2007). n.i.: non identified.

E.C. number	Name	Reaction	Gil *et. al.* 2004	Glass *et. al.* 2006
2.7.1.69	phosphotransferase system	glc + pep → g6p + pyr	PTS	MG041, 069, 429
5.3.1.9	glucose-6-phosphate isomerase	g6p ↔ f6p	PGI	MG111
2.7.1.11	6-phosphofructokinase	f6p + atp → fbp + adp	PFK	MG215
4.1.2.13	fructose-1,6-bisphosphate aldolase	fbp ↔ gdp + dhp	FBA	MG023
5.3.1.1	triose-phosphate isomerase	gdp ↔ dhp	TPI	MG431
1.2.1.12	glyceraldehyde-3-phosphate dehydrogenase	gdp + nad + p ↔ bpg + nadh	GAP	MG301
2.7.2.3	phosphoglycerate kinase	bpg + adp ↔ 3pg + atp	PGK	MG300
5.4.2.1	phosphoglycerate mutase	3pg ↔ 2pg	GPM	MG430
4.2.1.11	enolase	2pg ↔ pep	ENO	MG407
2.7.1.40	pyruvate kinase	pep + adp → pyr + atp	PYK	MG216
1.1.1.27	lactate dehydrogenase	pyr + nadh ↔ lac + nad	LDH	MG460
1.1.1.94	sn-glycerol-3-phosphate dehydrogenase	dhp + nadh → g3p + nad	GPS	n.i.
2.3.1.15	sn-glycerol-3-phosphate acyltransferase	g3p + pal → mag	PLSb	n.i.
2.3.1.51	1-acyl-sn-glycerol-3-phosphate	mag + pal → dag	PLSc	MG212

	acyltransferase			
2.7.7.41	phosphatidate cytidyltransferase	dag + ctp → cdp-dag + pp	CDS	MG437
2.7.8.8	phosphatidylserine synthase	cdp-dag + ser → pser + cmp	PSS	n.i.
4.1.1.65	phosphatidylserine decarboxylase	pser → peta	PSD	n.i.
4.1.2.13	fructose-1,6-bisphosphate aldolase	gdp + e4p ↔ sbp	FBA2	MG023
3.1.3.37	sedoheptulose-1,7-bisphosphatase	sbp → s7p + p	SPH	n.i.
2.2.1.1	transketolase	gdp + s7p ↔ rip + xip	TKT	MG066
2.2.1.1	transketolase	e4p + xip ↔ f6p + gdp	TKT2	MG066
5.1.3.1	ribulose-phosphate 3-epimerase	xip ↔ rup	RPE	MG112
5.3.1.6	ribose-5-phosphate isomerase	rup ↔ rip	RPI	MG396
2.7.6.1	phosphoribosylpyrophosphate synthetase	rip + atp → prpp + amp	PRS	MG058
2.4.2.8	hypoxanthine phosphoribosyltransferase	prpp + ade → amp + pp	HPT	MG276
2.4.2.8	hypoxanthine phosphoribosyltransferase	prpp + gua → gmp + pp	HPT2	MG458
2.4.2.9	uracil phosphoribosyltransferase	prpp + ura → ump + pp	UPP	MG030
3.6.1.1	inorganic pyrophosphatase	pp → 2p	PPA	MG351
2.7.4.3	adenylate kinase	amp + atp → 2adp	ADK	MG171
2.7.4.8	guanylate kinase	gmp + atp → gdp + adp	GMK	MG107
2.7.4.14	cytidylate kinase	ump + atp → udp + adp	CMK	MG330
2.7.4.14	cytidylate kinase	cmp + atp → cdp + adp	CMK2	MG330
2.7.4.6	nucleoside diphosphate kinase	gdp + atp ↔ gtp + adp	NDK	MG216

2.7.4.6	nucleoside diphosphate kinase	udp + atp ↔ utp + adp	NDK2	
2.7.4.6	nucleoside diphosphate kinase	dadp + atp ↔ datp + adp	NDK3	MG216
2.7.4.6	nucleoside diphosphate kinase	dgdp + atp ↔ dgtp + adp	NDK4	MG216
2.7.4.6	nucleoside diphosphate kinase	ctp + adp ↔ cdp + atp	NDK5	
2.7.4.6	nucleoside diphosphate kinase	dcdp + atp ↔ dctp + adp	NDK6	
2.7.4.6	nucleoside diphosphate kinase	dutp + adp ↔ dudp + atp	NDK7	
2.7.4.6	nucleoside diphosphate kinase	tdp + adp ↔ ttp + adp	NDK8	MG034
1.17.4.1	ribonucleoside diphosphate reductase	adp + nadh → dadp + nad	NRD	MG229–MG231
1.17.4.1	ribonucleoside diphosphate reductase	gdp + nadh → dgdp + nad	NRD2	MG229–MG231
1.17.4.1	ribonucleoside diphosphate reductase	cdp + nadh → dcdp + nad	NRD3	MG229–MG231
6.3.4.2	CTP synthase	utp → ctp	PYR	n.i.
3.5.4.13	dCTP deaminase	dctp → dutp	DCD	n.i.
2.7.4.9	thymidylate kinase	dudp + adp ↔ dump + atp	TMK	MG006
2.7.4.9	thymidylate kinase	tmp + atp ↔ tdp + adp	TMK2	MG006
2.1.1.45	thymidylate synthase	dump + mthf → dhf + tmp	THY	MG227
1.5.1.3	dihydrofolate reductase	dhf + nadh ↔ thf + nad	DFR	MG228
2.1.2.1	glycine hydroxymethyltransferase	ser + thf ↔ gly + mthf	GHT	MG394

Table S2. Reactions, and compounds that make up *Ca. Nasuia deltocephalinicola's* m-DAG. Reversible reactions are denoted by the superscript *r*.

Substrate KEGG id	ReactionID (E.C.number)	Definition	Product KEGG id
C00002	R00435^{r} (2.7.7.6)	ATP + RNA ↔ Diphosphate + RNA	C00046
C00131	R00375^{r} (2.7.7.7)	dATP + DNA ↔ Diphosphate + DNA	C00039
C00044	R00441^{r} (2.7.7.6)	GTP + RNA ↔ Diphosphate + RNA	C00046
C00286	R00376^{r} (2.7.7.7)	dGTP + DNA ↔ Diphosphate + DNA	C00039
C00075	R00443 (2.7.7.6)	UTP + RNA → Diphosphate + RNA	C00046
C00063	R00442 (2.7.7.6)	CTP + RNA → Diphosphate + RNA	C00046
C00458	R00377 (2.7.7.7)	dCTP + DNA → Diphosphate + DNA	C00039
C00459	R00378 (2.7.7.7)	dTTP + DNA → Diphosphate + DNA	C00039
C01118+C00283	R01288 (2.5.1.48)	O-Succinyl-L-homoserine + Hydrogen sulfide → L-Homocysteine + Succinate	C00155 + C00042
C00155	R04405 (2.1.1.14)	5-Methyltetrahydropteroyltri-L-glutamate + L-Homocysteine → Tetrahydropteroyltri-L-glutamate + L-Methionine	C00073
C02739	R01071^{r} (2.4.2.17)	1- (5-Phospho-D-ribosyl)-ATP + Diphosphate ↔ ATP + 5-Phospho-alpha-D-ribose 1-diphosphate	C00119
C01929	R01163 (1.1.1.23)	L-Histidinal + H2O + NAD+ → L-Histidine + NADH + H+	C00135
C00860	R03012 (1.1.1.23)	L-Histidinol + NAD+ → L-Histidinal + NADH + H+	C01929
C01100	R03243^{r} (2.6.1.9)	L-Histidinol phosphate + 2-Oxoglutarate ↔ 3- (Imidazol-4-yl)-2-oxopropyl phosphate + L-Glutamate	C01267
C04666	R03457 (4.2.1.19)	D-erythro-1- (Imidazol-4-yl)glycerol 3-phosphate → 3- (Imidazol-4-yl)-2-oxopropyl phosphate + H2O	C01267
C04896	R04640 (5.3.1.16)	5- (5-Phospho-D-ribosylaminoformimino)-1- (5-phosphoribosyl)-imidazole-4-carboxamide →N- (5'-Phospho-D-1'-ribulosylformimino)-5-amino-1- (5''-phospho-D-ribosyl)-4-imidazolecarboxamide	C04916
C02741	R04037 (3.5.4.19)	Phosphoribosyl-AMP + H2O → 5- (5-Phospho-D-ribosylaminoformimino)-1- (5-phosphoribosyl)-imidazole-4-carboxamide	C04916
C04916	R04558 (-.-.-.-)	N- (5'-Phospho-D-1'-ribulosylformimino)-5-amino-1- (5''-phospho-D-ribosyl)-4-imidazolecarboxamide + L-Glutamine → D-erythro-1- (Imidazol-4-yl)glycerol 3-phosphate + 1- (5'-Phosphoribosyl)-5-amino-4-imidazolecarboxamide + L-Glutamate	C04666 + C04677
C00166	R00694^{r} (2.6.1.9)	Phenylpyruvate + L-Glutamate ↔ L-Phenylalanine + 2-Oxoglutarate	C00079
C00082	R00734^{r} (2.6.1.9)	L-Tyrosine + 2-Oxoglutarate ↔ 3- (4-Hydroxyphenyl)pyruvate + L-Glutamate	C01179

C05698	R09365 (2.1.1.13|2.1.1.14)	Selenohomocysteine + 5-Methyltetrahydropteroyltri-L-glutamate → L-Selenomethionine + Tetrahydropteroyltri-L-glutamate	C05335
C18902	R09372 (1.8.1.9)	2 NADPH + 2 H+ + Methylselenic acid → 2 NADP+ + 2 H2O + Methaneselenol	C05703
C06148	R04639 (3.5.4.16)	2,5-Diamino-6- (5'-triphosphoryl-3',4'-trihydroxy-2'-oxopentyl)-amino-4-oxopyrimidine → 7,8-Dihydroneopterin 3'-triphosphate + H2O	C04895
C05923	R05048 (3.5.4.16)	2,5-Diaminopyrimidine nucleoside triphosphate → 2,5-Diamino-6- (5'-triphosphoryl-3',4'-trihydroxy-2'-oxopentyl)-amino-4-oxopyrimidine	C06148
C05922	R05046 (3.5.4.16)	Formamidopyrimidine nucleoside triphosphate + H2O → 2,5-Diaminopyrimidine nucleoside triphosphate + Formate	C05923
C00044	R00428 (3.5.4.16)	GTP + H2O → Formamidopyrimidine nucleoside triphosphate	C05922
C00283	R00858[r] (1.8.1.2)	Hydrogen sulfide + 3 NADP+ + 3 H2O ↔ Sulfite + 3 NADPH + 3 H+	C00094
C00053	R02021 (1.8.4.8)	Thioredoxin + 3'-Phosphoadenylyl sulfate → Thioredoxin disulfide + Sulfite + Adenosine 3',5'-bisphosphate	C00094
C00082	R02918 (6.1.1.1)	ATP + L-Tyrosine + tRNA (Tyr) → AMP + Diphosphate + L-Tyrosyl-tRNA (Tyr)	C02839
C02282	R03905 (6.3.5.7)	Glutaminyl-tRNA + L-Glutamate + Orthophosphate + ADP → L-Glutamyl-tRNA (Gln) + L-Glutamine + ATP + H2O	C06112
C03402	R04212 (6.3.5.6)	L-Asparaginyl-tRNA (Asn) + L-Glutamate + Orthophosphate + ADP ↔ L-Aspartyl-tRNA (Asn) + L-Glutamine + ATP + H2O	C06113

Table S3. Reactions included in the reconstruction of the JCVI-syn3.0 reaction graph and the minimal organism constructed for this work and the pathways in which each reaction (can) participates.

Reacción	JCVI-syn3.0	This work	Pathways in which they can participate
R02691(2.4.1.46)	Yes	No	1,2-diacylglycerol 3-beta-galactosyltransferase [EC:2.4.1.46]
R02241(2.3.1.51)	Yes	Yes	1-acyl-sn-glycerol-3-phosphate acyltransferase [EC:2.3.1.51]///lysophosphatidate acyltransferase [EC:2.3.1.51]///lysocardiolipin and lysophospholipid acyltransferase [EC:2.3.1.- 2.3.1.51]///lysophospholipid acyltransferase 1/2 [EC:2.3.1.51 2.3.1.-]///lysophospholipid acyltransferase [EC:2.3.1.51 2.3.1.23 2.3.1.-]///lysophosphatidic acid acyltransferase / lysophosphatidylinositol acyltransferase [EC:2.3.1.51 2.3.1.-]///TAG lipase / steryl ester hydrolase / phospholipase A2 / LPA acyltransferase [EC:3.1.1.3 3.1.1.13 3.1.1.4 2.3.1.51]///lysophosphatidiate acyltransferase [EC:2.3.1.51]///1-acylglycerol-3-phosphate O-acyltransferase [EC:2.3.1.51]
R00508(3.1.3.7)	Yes	No	3'(2'), 5'-bisphosphate nucleotidase [EC:3.1.3.7]///bifunctional oligoribonuclease and PAP phosphatase NrnA [EC:3.1.3.7 3.1.13.3]///3'(2'), 5'-bisphosphate nucleotidase / inositol polyphosphate 1-phosphatase [EC:3.1.3.7 3.1.3.57]///inositol monophosphatase 3 [EC:3.1.3.25 3.1.3.7]
R01968(3.1.3.5)	Yes	No	5'-nucleotidase [EC:3.1.3.5]///5'-nucleotidase [EC:3.1.3.5]///2',3'-cyclic-nucleotide 2'-phosphodiesterase / 3'-nucleotidase / 5'-nucleotidase [EC:3.1.4.16 3.1.3.6 3.1.3.5]///5'-deoxynucleotidase [EC:3.1.3.89]///5'-nucleotidase [EC:3.1.3.5]///5'-nucleotidase / UDP-sugar diphosphatase [EC:3.1.3.5 3.6.1.45]///5'-nucleotidase [EC:3.1.3.5]///5'-nucleotidase [EC:3.1.3.5]
R02088(3.1.3.5)	Yes	No	5'-nucleotidase [EC:3.1.3.5]///5'-nucleotidase [EC:3.1.3.5]///2',3'-cyclic-nucleotide 2'-phosphodiesterase / 3'-nucleotidase / 5'-nucleotidase [EC:3.1.4.16 3.1.3.6 3.1.3.5]///5'-deoxynucleotidase [EC:3.1.3.89]///5'-nucleotidase [EC:3.1.3.5]///5'-nucleotidase / UDP-sugar diphosphatase [EC:3.1.3.5 3.6.1.45]///5'-nucleotidase [EC:3.1.3.5]///5'-nucleotidase [EC:3.1.3.5]
R01569(3.1.3.5)	Yes	No	5'-nucleotidase [EC:3.1.3.5]///5'-nucleotidase [EC:3.1.3.5]///2',3'-cyclic-nucleotide 2'-phosphodiesterase / 3'-nucleotidase / 5'-nucleotidase [EC:3.1.4.16 3.1.3.6 3.1.3.5]///5'-deoxynucleotidase [EC:3.1.3.89]///5'-nucleotidase [EC:3.1.3.5]///5'-nucleotidase / UDP-sugar diphosphatase [EC:3.1.3.5 3.6.1.45]///5'-nucleotidase [EC:3.1.3.5]///5'-nucleotidase [EC:3.1.3.5]
R01664(3.1.3.5)	Yes	No	5'-nucleotidase [EC:3.1.3.5]///5'-nucleotidase [EC:3.1.3.5]///2',3'-cyclic-nucleotide 2'-phosphodiesterase / 3'-nucleotidase / 5'-nucleotidase [EC:3.1.4.16 3.1.3.6 3.1.3.5]///5'-deoxynucleotidase [EC:3.1.3.89]///5'-nucleotidase [EC:3.1.3.5]///5'-nucleotidase / UDP-sugar diphosphatase [EC:3.1.3.5 3.6.1.45]///5'-nucleotidase [EC:3.1.3.5]///5'-nucleotidase [EC:3.1.3.5]
R02102(3.1.3.5)	Yes	No	5'-nucleotidase [EC:3.1.3.5]///5'-nucleotidase [EC:3.1.3.5]///2',3'-cyclic-nucleotide 2'-phosphodiesterase / 3'-nucleotidase / 5'-nucleotidase [EC:3.1.4.16 3.1.3.6 3.1.3.5]///5'-deoxynucleotidase [EC:3.1.3.89]///5'-nucleotidase [EC:3.1.3.5]///5'-nucleotidase / UDP-sugar diphosphatase [EC:3.1.3.5 3.6.1.45]///5'-nucleotidase [EC:3.1.3.5]///5'-nucleotidase [EC:3.1.3.5]
R00183(3.1.3.5)	Yes	No	5'-nucleotidase [EC:3.1.3.5]///5'-nucleotidase [EC:3.1.3.5]///2',3'-cyclic-nucleotide 2'-phosphodiesterase / 3'-nucleotidase / 5'-nucleotidase [EC:3.1.4.16 3.1.3.6 3.1.3.5]///5'-nucleotidase [EC:3.1.3.5]///5'-nucleotidase / UDP-sugar diphosphatase [EC:3.1.3.5 3.6.1.45]///5'-nucleotidase [EC:3.1.3.5]///5'-nucleotidase [EC:3.1.3.5]
R01126(3.1.3.5)	Yes	No	5'-nucleotidase [EC:3.1.3.5]///5'-nucleotidase [EC:3.1.3.5]///2',3'-cyclic-nucleotide 2'-phosphodiesterase / 3'-nucleotidase / 5'-nucleotidase [EC:3.1.4.16 3.1.3.6 3.1.3.5]///5'-nucleotidase [EC:3.1.3.5]///5'-nucleotidase / UDP-sugar diphosphatase [EC:3.1.3.5 3.6.1.45]///IMP and pyridine-specific 5'-nucleotidase [EC:3.1.3.99 3.1.3.-]///5'-nucleotidase [EC:3.1.3.5]///5'-nucleotidase [EC:3.1.3.5]

R01227(3.1.3.5)	Yes	No	5'-nucleotidase [EC:3.1.3.5]///5'-nucleotidase [EC:3.1.3.5]///2',3'-cyclic-nucleotide 2'-phosphodiesterase / 3'-nucleotidase / 5'-nucleotidase [EC:3.1.4.16 3.1.3.6 3.1.3.5]///5'-nucleotidase [EC:3.1.3.5]///5'-nucleotidase / UDP-sugar diphosphatase [EC:3.1.3.5 3.6.1.45]///5'-nucleotidase [EC:3.1.3.5]///5'-nucleotidase [EC:3.1.3.5]
R02719(3.1.3.5)	Yes	No	5'-nucleotidase [EC:3.1.3.5]///5'-nucleotidase [EC:3.1.3.5]///2',3'-cyclic-nucleotide 2'-phosphodiesterase / 3'-nucleotidase / 5'-nucleotidase [EC:3.1.4.16 3.1.3.6 3.1.3.5]///5'-nucleotidase [EC:3.1.3.5]///5'-nucleotidase / UDP-sugar diphosphatase [EC:3.1.3.5 3.6.1.45]///5'-nucleotidase [EC:3.1.3.5]///5'-nucleotidase [EC:3.1.3.5]
R00511(3.1.3.5)	Yes	No	5'-nucleotidase [EC:3.1.3.5]///5'-nucleotidase [EC:3.1.3.5]///2',3'-cyclic-nucleotide 2'-phosphodiesterase / 3'-nucleotidase / 5'-nucleotidase [EC:3.1.4.16 3.1.3.6 3.1.3.5]///5'-nucleotidase [EC:3.1.3.5]///5'-nucleotidase / UDP-sugar diphosphatase [EC:3.1.3.5 3.6.1.45]///5'-nucleotidase [EC:3.1.3.5]///5'-nucleotidase [EC:3.1.3.5]
R00963(3.1.3.5)	Yes	No	5'-nucleotidase [EC:3.1.3.5]///5'-nucleotidase [EC:3.1.3.5]///2',3'-cyclic-nucleotide 2'-phosphodiesterase / 3'-nucleotidase / 5'-nucleotidase [EC:3.1.4.16 3.1.3.6 3.1.3.5]///5'-nucleotidase [EC:3.1.3.5]///5'-nucleotidase / UDP-sugar diphosphatase [EC:3.1.3.5 3.6.1.45]///5'-nucleotidase [EC:3.1.3.5]///5'-nucleotidase [EC:3.1.3.5]
R02323(3.1.3.5)	Yes	No	5'-nucleotidase [EC:3.1.3.5]///5'-nucleotidase [EC:3.1.3.5]///5'-nucleotidase [EC:3.1.3.5]///5'-nucleotidase / UDP-sugar diphosphatase [EC:3.1.3.5 3.6.1.45]///IMP and pyridine-specific 5'-nucleotidase [EC:3.1.3.99 3.1.3.-]///pyrimidine and pyridine-specific 5'-nucleotidase [EC:3.1.3.-]///5'-nucleotidase [EC:3.1.3.5]///5'-nucleotidase [EC:3.1.3.5]
R03346(3.1.3.5)	Yes	No	5'-nucleotidase [EC:3.1.3.5]///5'-nucleotidase [EC:3.1.3.5]///5'-nucleotidase [EC:3.1.3.5]///5'-nucleotidase / UDP-sugar diphosphatase [EC:3.1.3.5 3.6.1.45]///IMP and pyridine-specific 5'-nucleotidase [EC:3.1.3.99 3.1.3.-]///pyrimidine and pyridine-specific 5'-nucleotidase [EC:3.1.3.-]///5'-nucleotidase [EC:3.1.3.5]
R01569(3.1.3.89)	Yes	No	5'-nucleotidase [EC:3.1.3.5]///5'-nucleotidase [EC:3.1.3.5]///2',3'-cyclic-nucleotide 2'-phosphodiesterase / 3'-nucleotidase / 5'-nucleotidase [EC:3.1.4.16 3.1.3.6 3.1.3.5]///5'-deoxynucleotidase [EC:3.1.3.89]///5'-nucleotidase [EC:3.1.3.5]///5'-nucleotidase / UDP-sugar diphosphatase [EC:3.1.3.5 3.6.1.45]///5'-nucleotidase [EC:3.1.3.5]///5'-nucleotidase [EC:3.1.3.5]
R01664(3.1.3.89)	Yes	No	5'-nucleotidase [EC:3.1.3.5]///5'-nucleotidase [EC:3.1.3.5]///2',3'-cyclic-nucleotide 2'-phosphodiesterase / 3'-nucleotidase / 5'-nucleotidase [EC:3.1.4.16 3.1.3.6 3.1.3.5]///5'-deoxynucleotidase [EC:3.1.3.89]///5'-nucleotidase [EC:3.1.3.5]///5'-nucleotidase / UDP-sugar diphosphatase [EC:3.1.3.5 3.6.1.45]///5'-nucleotidase [EC:3.1.3.5]///5'-nucleotidase [EC:3.1.3.5]
R02102(3.1.3.89)	Yes	No	5'-nucleotidase [EC:3.1.3.5]///5'-nucleotidase [EC:3.1.3.5]///2',3'-cyclic-nucleotide 2'-phosphodiesterase / 3'-nucleotidase / 5'-nucleotidase [EC:3.1.4.16 3.1.3.6 3.1.3.5]///5'-deoxynucleotidase [EC:3.1.3.89]///5'-nucleotidase [EC:3.1.3.5]///5'-nucleotidase / UDP-sugar diphosphatase [EC:3.1.3.5 3.6.1.45]///5'-nucleotidase [EC:3.1.3.5]///5'-nucleotidase [EC:3.1.3.5]
R04779(2.7.1.11)	Yes	Yes	6-phosphofructokinase 1 [EC:2.7.1.11]///6-phosphofructokinase 2 [EC:2.7.1.11]///ATP-dependent phosphofructokinase / diphosphate-dependent phosphofructokinase [EC:2.7.1.11 2.7.1.90]///6-phosphofructokinase [EC:2.7.1.11]
R00315(2.7.2.1)	Yes	No	acetate kinase [EC:2.7.2.1]
R01353(2.7.2.1)	Yes	No	acetate kinase [EC:2.7.2.1]///propionate kinase [EC:2.7.2.15]///propionate kinase [EC:2.7.2.15]
R04378(2.4.2.7)	Yes	No	adenine phosphoribosyltransferase [EC:2.4.2.7]
R00190(2.4.2.7)	Yes	Yes	adenine phosphoribosyltransferase [EC:2.4.2.7]///hypoxanthine phosphoribosyltransferase [EC:2.4.2.8]
R01229(2.4.2.7)	Yes	Yes	adenine phosphoribosyltransferase [EC:2.4.2.7]///hypoxanthine phosphoribosyltransferase [EC:2.4.2.8]///xanthine phosphoribosyltransferase [EC:2.4.2.22]///xanthine phosphoribosyltransferase [EC:2.4.2.22]///bifunctional protein

			TilS/HprT [EC:6.3.4.19 2.4.2.8]///hypoxanthine phosphoribosyltransferase [EC:2.4.2.8]
R01229(2.4.2.8)	Yes	Yes	adenine phosphoribosyltransferase [EC:2.4.2.7]///hypoxanthine phosphoribosyltransferase [EC:2.4.2.8]///xanthine phosphoribosyltransferase [EC:2.4.2.22]///xanthine phosphoribosyltransferase [EC:2.4.2.22]///bifunctional protein TilS/HprT [EC:6.3.4.19 2.4.2.8]///hypoxanthine phosphoribosyltransferase [EC:2.4.2.8]
R00185(2.7.1.74)	Yes	No	adenosine kinase [EC:2.7.1.20]///deoxycitidine kinase [EC:2.7.1.74]///deoxyadenosine/deoxycytidine kinase [EC:2.7.1.76 2.7.1.74]
R00086(3.6.1.15)	Yes	No	adenosinetriphosphatase [EC:3.6.1.3]///apyrase [EC:3.6.1.5]///nucleoside triphosphate diphosphatase [EC:3.6.1.9]///nucleoside-triphosphatase [EC:3.6.1.15]///apyrase [EC:3.6.1.5]///golgi apyrase [EC:3.6.1.5]
R11319(2.7.4.3)	Yes	No	adenylate kinase [EC:2.7.4.3]
R01547(2.7.4.11)	Yes	No	adenylate kinase [EC:2.7.4.3]///adenylate kinase [EC:2.7.4.3]///adenylate/nucleoside-diphosphate kinase [EC:2.7.4.3 2.7.4.6]
R01547(2.7.4.3)	Yes	No	adenylate kinase [EC:2.7.4.3]///adenylate kinase [EC:2.7.4.3]///adenylate/nucleoside-diphosphate kinase [EC:2.7.4.3 2.7.4.6]
R01801(2.7.8.5)	Yes	No	CDP-diacylglycerol---glycerol-3-phosphate 3-phosphatidyltransferase [EC:2.7.8.5]
R01800(2.7.8.8)	No	Yes	CDP-diacylglycerol---serine O-phosphatidyltransferase [EC:2.7.8.8]///CDP-diacylglycerol---serine O-phosphatidyltransferase [EC:2.7.8.8]
R00512(2.7.4.25)	Yes	Yes	CMP/dCMP kinase [EC:2.7.4.25]///pantoate ligase / CMP/dCMP kinase [EC:6.3.2.1 2.7.4.25]///UMP-CMP kinase [EC:2.7.4.14]///UMP-CMP kinase 2, mitochondrial [EC:2.7.4.14]
R01665(2.7.4.25)	Yes	No	CMP/dCMP kinase [EC:2.7.4.25]///pantoate ligase / CMP/dCMP kinase [EC:6.3.2.1 2.7.4.25]///UMP-CMP kinase [EC:2.7.4.14]///UMP-CMP kinase 2, mitochondrial [EC:2.7.4.14]
R00158(2.7.4.22)	Yes	Yes	CMP/dCMP kinase [EC:2.7.4.25]///uridylate kinase [EC:2.7.4.22]///pantoate ligase / CMP/dCMP kinase [EC:6.3.2.1 2.7.4.25]///UMP-CMP kinase [EC:2.7.4.14]///UMP-CMP kinase 2, mitochondrial [EC:2.7.4.14]
R00571(6.3.4.2)	Yes	Yes	CTP synthase [EC:6.3.4.2]
R00573(6.3.4.2)	Yes	Yes	CTP synthase [EC:6.3.4.2]
R01663(3.5.4.12)	Yes	No	dCMP deaminase [EC:3.5.4.12]
R02325(3.5.4.13)	No	Yes	dCTP deaminase [EC:3.5.4.13]
R01667(3.6.1.12)	Yes	No	dCTP diphosphatase [EC:3.6.1.12]
R01668(3.6.1.12)	Yes	No	dCTP diphosphatase [EC:3.6.1.12]
R02089(2.7.1.76)	Yes	No	deoxyadenosine kinase [EC:2.7.1.76]///deoxyadenosine/deoxycytidine kinase [EC:2.7.1.76 2.7.1.74]
R01666(2.7.1.74)	Yes	No	deoxycitidine kinase [EC:2.7.1.74]///deoxyadenosine/deoxycytidine kinase [EC:2.7.1.76 2.7.1.74]///cytidine kinase [EC:2.7.1.213]
R01967(2.7.1.113)	Yes	No	deoxyguanosine kinase [EC:2.7.1.113]///deoxyguanosine kinase [EC:2.7.1.113]
R02235(1.5.1.3)	No	Yes	dihydrofolate reductase [EC:1.5.1.3]///dihydrofolate reductase / thymidylate synthase [EC:1.5.1.3 2.1.1.45]///dihydrofolate

			reductase (trimethoprim resistance protein) [EC:1.5.1.3]///dihydrofolate reductase (trimethoprim resistance protein) [EC:1.5.1.3]///dihydrofolate reductase (trimethoprim resistance protein) [EC:1.5.1.3]///dihydrofolate reductase (trimethoprim resistance protein) [EC:1.5.1.3]///dihydrofolate reductase (trimethoprim resistance protein) [EC:1.5.1.3]
R02094(2.7.4.9)	Yes	Yes	dTMP kinase [EC:2.7.4.9]
R02098(2.7.4.9)	Yes	Yes	dTMP kinase [EC:2.7.4.9]
R11896(3.6.1.23)	Yes	No	dUTP pyrophosphatase [EC:3.6.1.23]
R00161(2.7.7.2)	Yes	No	FAD synthetase [EC:2.7.7.2]///riboflavin kinase / FMN adenylyltransferase [EC:2.7.1.26 2.7.7.2]///FAD synthetase [EC:2.7.7.2]///FAD synthetase [EC:2.7.7.2]
R00942(6.3.2.17)	Yes	No	folylpolyglutamate synthase [EC:6.3.2.17]///dihydrofolate synthase / folylpolyglutamate synthase [EC:6.3.2.12 6.3.2.17]///dihydrofolate synthase / folylpolyglutamate synthase [EC:6.3.2.12 6.3.2.17]
R04241(6.3.2.17)	Yes	No	folylpolyglutamate synthase [EC:6.3.2.17]///dihydrofolate synthase / folylpolyglutamate synthase [EC:6.3.2.12 6.3.2.17]///dihydrofolate synthase / folylpolyglutamate synthase [EC:6.3.2.12 6.3.2.17]
R02237(6.3.2.17)	Yes	No	folylpolyglutamate synthase [EC:6.3.2.17]///dihydrofolate synthase / folylpolyglutamate synthase [EC:6.3.2.12 6.3.2.17]///dihydrofolate synthase [EC:6.3.2.12]///dihydrofolate synthase / folylpolyglutamate synthase [EC:6.3.2.12 6.3.2.17]///dihydrofolate synthase / dihydropteroate synthase [EC:6.3.2.12 2.5.1.15]
R00765(3.5.99.6)	Yes	No	glucosamine-6-phosphate deaminase [EC:3.5.99.6]
R02301(6.3.3.2)	Yes	No	glutamate formiminotransferase / 5-formyltetrahydrofolate cyclo-ligase [EC:2.1.2.5 6.3.3.2]///5-formyltetrahydrofolate cyclo-ligase [EC:6.3.3.2]
R01058(1.2.1.9)	Yes	No	glyceraldehyde-3-phosphate dehydrogenase (NADP+) [EC:1.2.1.9]///glyceraldehyde-3-phosphate dehydrogenase [NAD(P)+] [EC:1.2.1.90]
R00847(2.7.1.30)	Yes	No	glycerol kinase [EC:2.7.1.30]
R00851(2.3.1.15)	No	Yes	glycerol-3-phosphate O-acyltransferase 1/2 [EC:2.3.1.15]///glycerol-3-phosphate O-acyltransferase [EC:2.3.1.15]///glycerol-3-phosphate O-acyltransferase [EC:2.3.1.15]///glycerol-3-phosphate O-acyltransferase 3/4 [EC:2.3.1.15]///glycerol-3-phosphate O-acyltransferase / dihydroxyacetone phosphate acyltransferase [EC:2.3.1.15 2.3.1.42]///glycerol-3-phosphate acyltransferase [EC:2.3.1.15 2.3.1.198]
R00945(2.1.2.1)	Yes	Yes	glycine hydroxymethyltransferase [EC:2.1.2.1]
R09099(2.1.2.1)	Yes	No	glycine hydroxymethyltransferase [EC:2.1.2.1]
R00332(2.7.4.8)	Yes	Yes	guanylate kinase [EC:2.7.4.8]
R02090(2.7.4.8)	Yes	No	guanylate kinase [EC:2.7.4.8]
R01625(2.7.8.7)	Yes	No	holo-[acyl-carrier protein] synthase [EC:2.7.8.7]///4'-phosphopantetheinyl transferase [EC:2.7.8.-]
R08237(2.4.2.8)	Yes	No	hypoxanthine phosphoribosyltransferase [EC:2.4.2.8]
R08238(2.4.2.8)	Yes	No	hypoxanthine phosphoribosyltransferase [EC:2.4.2.8]

R08245(2.4.2.8)	Yes	No	hypoxanthine phosphoribosyltransferase [EC:2.4.2.8]
R01132(2.4.2.8)	Yes	No	hypoxanthine phosphoribosyltransferase [EC:2.4.2.8]///bifunctional protein TilS/HprT [EC:6.3.4.19 2.4.2.8]///hypoxanthine phosphoribosyltransferase [EC:2.4.2.8]
R02142(2.4.2.8)	Yes	No	hypoxanthine phosphoribosyltransferase [EC:2.4.2.8]///xanthine phosphoribosyltransferase [EC:2.4.2.22]///xanthine phosphoribosyltransferase [EC:2.4.2.22]///bifunctional protein TilS/HprT [EC:6.3.4.19 2.4.2.8]///hypoxanthine phosphoribosyltransferase [EC:2.4.2.8]
R02100(3.6.1.23)	Yes	No	inosine triphosphate pyrophosphatase [EC:3.6.1.-]///dUTP pyrophosphatase [EC:3.6.1.23]///XTP/dITP diphosphohydrolase [EC:3.6.1.66]
R00703(1.1.1.27)	No	Yes	L-lactate dehydrogenase [EC:1.1.1.27]
R03940(2.1.2.9)	Yes	No	methionyl-tRNA formyltransferase [EC:2.1.2.9]
R01655(3.5.4.9)	Yes	No	methylenetetrahydrofolate dehydrogenase (NADP+) / methenyltetrahydrofolate cyclohydrolase / formyltetrahydrofolate synthetase [EC:1.5.1.5 3.5.4.9 6.3.4.3]///methylenetetrahydrofolate dehydrogenase (NADP+) / methenyltetrahydrofolate cyclohydrolase [EC:1.5.1.5 3.5.4.9]///methenyltetrahydrofolate cyclohydrolase [EC:3.5.4.9]///methylenetetrahydrofolate dehydrogenase(NAD+) / 5,10-methenyltetrahydrofolate cyclohydrolase [EC:1.5.1.15 3.5.4.9]
R01220(1.5.1.5)	Yes	No	methylenetetrahydrofolate dehydrogenase (NADP+) / methenyltetrahydrofolate cyclohydrolase / formyltetrahydrofolate synthetase [EC:1.5.1.5 3.5.4.9 6.3.4.3]///methylenetetrahydrofolate/methylenetetrahydromethanopterin dehydrogenase (NADP+) [EC:1.5.1.5 1.5.1.-]///methylenetetrahydrofolate dehydrogenase (NADP+) / methenyltetrahydrofolate cyclohydrolase [EC:1.5.1.5 3.5.4.9]
R05168(3.5.1.25)	Yes	No	N-acetylgalactosamine-6-phosphate deacetylase [EC:3.5.1.25]
R02059(3.5.1.25)	Yes	No	N-acetylglucosamine-6-phosphate deacetylase [EC:3.5.1.25]
R02705(2.7.1.60)	Yes	No	N-acylmannosamine kinase [EC:2.7.1.60]///bifunctional UDP-N-acetylglucosamine 2-epimerase / N-acetylmannosamine kinase [EC:3.2.1.183 2.7.1.60]///N-acetylmannosamine-6-phosphate 2-epimerase / N-acetylmannosamine kinase [EC:5.1.3.9 2.7.1.60]
R00104(2.7.1.23)	Yes	No	NAD+ kinase [EC:2.7.1.23]
R00189(6.3.1.5)	Yes	No	NAD+ synthase [EC:6.3.1.5]
R01271(2.4.2.12)	Yes	No	nicotinamide phosphoribosyltransferase [EC:2.4.2.12]
R00137(2.7.7.18)	Yes	No	nicotinamide-nucleotide adenylyltransferase [EC:2.7.7.1]///nicotinate-nucleotide adenylyltransferase [EC:2.7.7.18]///nicotinamide mononucleotide adenylyltransferase [EC:2.7.7.1 2.7.7.18]///HTH-type transcriptional regulator, transcriptional repressor of NAD biosynthesis genes [EC:2.7.7.1 2.7.1.22]///bifunctional NMN adenylyltransferase/nudix hydrolase [EC:2.7.7.1 3.6.1.-]///nicotinamide-nucleotide adenylyltransferase [EC:2.7.7.1]
R03005(2.7.7.18)	Yes	No	nicotinamide-nucleotide adenylyltransferase [EC:2.7.7.1]///nicotinate-nucleotide adenylyltransferase [EC:2.7.7.18]///nicotinamide mononucleotide adenylyltransferase [EC:2.7.7.1 2.7.7.18]///HTH-type transcriptional regulator, transcriptional repressor of NAD biosynthesis genes [EC:2.7.7.1 2.7.1.22]///bifunctional NMN adenylyltransferase/nudix hydrolase [EC:2.7.7.1 3.6.1.-]///nicotinamide-nucleotide adenylyltransferase [EC:2.7.7.1]

R00615(3.6.1.15)	Yes	No	nucleoside-triphosphatase [EC:3.6.1.15]///ribosome biogenesis GTPase / thiamine phosphate phosphatase [EC:3.6.1.- 3.1.3.100]
R00921(2.3.1.8)	Yes	No	phosphate acetyltransferase [EC:2.3.1.8]///phosphate acetyltransferase [EC:2.3.1.8]///phosphate propanoyltransferase [EC:2.3.1.222]///putative phosphotransacetylase [EC:2.3.1.8]
R00230(2.3.1.8)	Yes	No	phosphate acetyltransferase [EC:2.3.1.8]///phosphotransacetylase///phosphate acetyltransferase [EC:2.3.1.8]///putative phosphotransacetylase [EC:2.3.1.8]
R01799(2.7.7.41)	Yes	Yes	phosphatidate cytidylyltransferase [EC:2.7.7.41]
R04162(3.1.3.4)	Yes	No	phosphatidate phosphatase [EC:3.1.3.4]
R02029(3.1.3.27)	Yes	No	phosphatidylglycerophosphatase GEP4 [EC:3.1.3.27]///phosphatidylglycerophosphatase A [EC:3.1.3.27]///phosphatidylglycerophosphatase B [EC:3.1.3.27 3.1.3.81 3.1.3.4 3.6.1.27]///phosphatidylglycerophosphatase C [EC:3.1.3.27]
R01512(2.7.2.3)	Yes	Yes	phosphoglycerate kinase [EC:2.7.2.3]
R01969(2.4.2.1)	Yes	No	purine-nucleoside phosphorylase [EC:2.4.2.1]///purine-nucleoside phosphorylase [EC:2.4.2.1]
R02557(2.4.2.1)	Yes	No	purine-nucleoside phosphorylase [EC:2.4.2.1]///purine-nucleoside phosphorylase [EC:2.4.2.1]
R02748(2.4.2.1)	Yes	No	purine-nucleoside phosphorylase [EC:2.4.2.1]///purine-nucleoside phosphorylase [EC:2.4.2.1]
R02294(2.4.2.1)	Yes	No	purine-nucleoside phosphorylase [EC:2.4.2.1]///purine-nucleoside phosphorylase [EC:2.4.2.1]
R02295(2.4.2.1)	Yes	No	purine-nucleoside phosphorylase [EC:2.4.2.1]///purine-nucleoside phosphorylase [EC:2.4.2.1]
R01561(2.4.2.1)	Yes	No	purine-nucleoside phosphorylase [EC:2.4.2.1]///purine-nucleoside phosphorylase [EC:2.4.2.1]///purine/pyrimidine-nucleoside phosphorylase [EC:2.4.2.1 2.4.2.2]
R01863(2.4.2.1)	Yes	No	purine-nucleoside phosphorylase [EC:2.4.2.1]///purine-nucleoside phosphorylase [EC:2.4.2.1]///purine/pyrimidine-nucleoside phosphorylase [EC:2.4.2.1 2.4.2.2]
R02147(2.4.2.1)	Yes	No	purine-nucleoside phosphorylase [EC:2.4.2.1]///purine-nucleoside phosphorylase [EC:2.4.2.1]///purine/pyrimidine-nucleoside phosphorylase [EC:2.4.2.1 2.4.2.2]
R02297(2.4.2.1)	Yes	No	purine-nucleoside phosphorylase [EC:2.4.2.1]///purine-nucleoside phosphorylase [EC:2.4.2.1]///xanthosine phosphorylase [EC:2.4.2.-]///purine/pyrimidine-nucleoside phosphorylase [EC:2.4.2.1 2.4.2.2]
R02484(2.4.2.1)	Yes	No	pyrimidine-nucleoside phosphorylase [EC:2.4.2.2]///uridine phosphorylase [EC:2.4.2.3]///thymidine phosphorylase [EC:2.4.2.4]///purine-nucleoside phosphorylase [EC:2.4.2.1]///purine-nucleoside phosphorylase [EC:2.4.2.1]
R00200(2.7.1.40)	Yes	Yes	pyruvate kinase [EC:2.7.1.40]///pyruvate kinase isozymes R/L [EC:2.7.1.40]
R00430(2.7.1.40)	Yes	Yes	pyruvate kinase [EC:2.7.1.40]///pyruvate kinase isozymes R/L [EC:2.7.1.40]
R01138(2.7.1.40)	Yes	Yes	pyruvate kinase [EC:2.7.1.40]///pyruvate kinase isozymes R/L [EC:2.7.1.40]
R01858(2.7.1.40)	Yes	Yes	pyruvate kinase [EC:2.7.1.40]///pyruvate kinase isozymes R/L [EC:2.7.1.40]

R00549(2.7.1.26)	Yes	No	riboflavin kinase [EC:2.7.1.26]///riboflavin kinase / FMN adenylyltransferase [EC:2.7.1.26 2.7.7.2]///riboflavin kinase / FMN hydrolase [EC:2.7.1.26 3.1.3.102]
R08363(1.17.4.1)	Yes	No	ribonucleoside-diphosphate reductase subunit M1 [EC:1.17.4.1]///ribonucleoside-diphosphate reductase subunit M2 [EC:1.17.4.1]
R11893(1.17.4.1)	Yes	No	ribonucleoside-diphosphate reductase subunit M1 [EC:1.17.4.1]///ribonucleoside-diphosphate reductase subunit M2 [EC:1.17.4.1]
R02018(1.17.4.1)	Yes	No	ribonucleotide reductase, class II [EC:1.17.4.1]///ribonucleoside-diphosphate reductase alpha chain [EC:1.17.4.1]///ribonucleoside-diphosphate reductase beta chain [EC:1.17.4.1]
R02017(1.17.4.1)	Yes	Yes	ribonucleotide reductase, class II [EC:1.17.4.1]///ribonucleoside-diphosphate reductase alpha chain [EC:1.17.4.1]///ribonucleoside-diphosphate reductase beta chain [EC:1.17.4.1]///ribonucleoside-diphosphate reductase subunit M1 [EC:1.17.4.1]///ribonucleoside-diphosphate reductase subunit M2 [EC:1.17.4.1]
R02019(1.17.4.1)	Yes	Yes	ribonucleotide reductase, class II [EC:1.17.4.1]///ribonucleoside-diphosphate reductase alpha chain [EC:1.17.4.1]///ribonucleoside-diphosphate reductase beta chain [EC:1.17.4.1]///ribonucleoside-diphosphate reductase subunit M1 [EC:1.17.4.1]///ribonucleoside-diphosphate reductase subunit M2 [EC:1.17.4.1]
R02024(1.17.4.1)	Yes	Yes	ribonucleotide reductase, class II [EC:1.17.4.1]///ribonucleoside-diphosphate reductase alpha chain [EC:1.17.4.1]///ribonucleoside-diphosphate reductase beta chain [EC:1.17.4.1]///ribonucleoside-diphosphate reductase subunit M1 [EC:1.17.4.1]///ribonucleoside-diphosphate reductase subunit M2 [EC:1.17.4.1]
R01049(2.7.6.1)	Yes	Yes	ribose-phosphate pyrophosphokinase [EC:2.7.6.1]
R07618(1.8.1.4)	Yes	No	rn00010: Glycolysis / Gluconeogenesis///rn00020: Citrate cycle (TCA cycle)///rn00280: Valine, leucine and isoleucine degradation///rn00620: Pyruvate metabolism///rn00640: Propanoate metabolism///rn01100: Metabolic pathways///rn01110: Biosynthesis of secondary metabolites
R03270(1.2.4.1)	Yes	No	rn00010: Glycolysis / Gluconeogenesis///rn00020: Citrate cycle (TCA cycle)///rn00620: Pyruvate metabolism
R00014(1.2.4.1)	Yes	No	rn00010: Glycolysis / Gluconeogenesis///rn00020: Citrate cycle (TCA cycle)///rn00620: Pyruvate metabolism
R02569(2.3.1.12)	Yes	No	rn00010: Glycolysis / Gluconeogenesis///rn00020: Citrate cycle (TCA cycle)///rn00620: Pyruvate metabolism
R01070(4.1.2.13)	Yes	Yes	rn00010: Glycolysis / Gluconeogenesis///rn00030: Pentose phosphate pathway///rn00051: Fructose and mannose metabolism///rn01100: Metabolic pathways///rn01110: Biosynthesis of secondary metabolites///rn01120: Microbial metabolism in diverse environments///rn01200: Carbon metabolism///rn01230: Biosynthesis of amino acids
R02740(5.3.1.9)	Yes	Yes	rn00010: Glycolysis / Gluconeogenesis///rn00030: Pentose phosphate pathway///rn00520: Amino sugar and nucleotide sugar metabolism///rn01100: Metabolic pathways///rn01110: Biosynthesis of secondary metabolites///rn01120: Microbial metabolism in diverse environments///rn01200: Carbon metabolism
R02739(5.3.1.9)	Yes	No	rn00010: Glycolysis / Gluconeogenesis///rn00030: Pentose phosphate pathway///rn01100: Metabolic pathways///rn01110: Biosynthesis of secondary metabolites///rn01120: Microbial metabolism in diverse environments
R01015(5.3.1.1)	Yes	No	rn00010: Glycolysis / Gluconeogenesis///rn00051: Fructose and mannose metabolism///rn00562: Inositol phosphate metabolism///rn00710: Carbon fixation in photosynthetic organisms///rn01100: Metabolic pathways///rn01110: Biosynthesis of secondary metabolites///rn01120: Microbial metabolism in diverse environments///rn01200: Carbon

			metabolism///rn01230: Biosynthesis of amino acids
R00959(5.4.2.5)	Yes	No	rn00010: Glycolysis / Gluconeogenesis///rn00052: Galactose metabolism///rn00520: Amino sugar and nucleotide sugar metabolism///rn01100: Metabolic pathways///rn01110: Biosynthesis of secondary metabolites///rn01120: Microbial metabolism in diverse environments
R01518(5.4.2.12)	Yes	Yes	rn00010: Glycolysis / Gluconeogenesis///rn00260: Glycine, serine and threonine metabolism///rn00680: Methane metabolism///rn01100: Metabolic pathways///rn01110: Biosynthesis of secondary metabolites///rn01120: Microbial metabolism in diverse environments///rn01200: Carbon metabolism///rn01230: Biosynthesis of amino acids
R00658(4.2.1.11)	Yes	Yes	rn00010: Glycolysis / Gluconeogenesis///rn00680: Methane metabolism///rn01100: Metabolic pathways///rn01110: Biosynthesis of secondary metabolites///rn01120: Microbial metabolism in diverse environments///rn01200: Carbon metabolism///rn01230: Biosynthesis of amino acids
R01061(1.2.1.12)	Yes	Yes	rn00010: Glycolysis / Gluconeogenesis///rn00710: Carbon fixation in photosynthetic organisms///rn01100: Metabolic pathways///rn01110: Biosynthesis of secondary metabolites///rn01120: Microbial metabolism in diverse environments///rn01200: Carbon metabolism///rn01230: Biosynthesis of amino acids
R03321(5.3.1.9)	Yes	No	rn00010: Glycolysis / Gluconeogenesis///rn01100: Metabolic pathways///rn01110: Biosynthesis of secondary metabolites///rn01120: Microbial metabolism in diverse environments
R01529(5.1.3.1)	Yes	Yes	rn00030: Pentose phosphate pathway///rn00040: Pentose and glucuronate interconversions///rn00710: Carbon fixation in photosynthetic organisms///rn01100: Metabolic pathways///rn01110: Biosynthesis of secondary metabolites///rn01120: Microbial metabolism in diverse environments///rn01200: Carbon metabolism///rn01230: Biosynthesis of amino acids
R01057(5.4.2.7)	Yes	No	rn00030: Pentose phosphate pathway///rn00230: Purine metabolism///rn01100: Metabolic pathways
R01641(2.2.1.1)	Yes	Yes	rn00030: Pentose phosphate pathway///rn00710: Carbon fixation in photosynthetic organisms///rn01100: Metabolic pathways///rn01110: Biosynthesis of secondary metabolites///rn01120: Microbial metabolism in diverse environments///rn01200: Carbon metabolism///rn01230: Biosynthesis of amino acids
R01056(5.3.1.6)	Yes	Yes	rn00030: Pentose phosphate pathway///rn00710: Carbon fixation in photosynthetic organisms///rn01100: Metabolic pathways///rn01110: Biosynthesis of secondary metabolites///rn01120: Microbial metabolism in diverse environments///rn01200: Carbon metabolism///rn01230: Biosynthesis of amino acids
R01066(4.1.2.4)	Yes	No	rn00030: Pentose phosphate pathway///rn01100: Metabolic pathways
R02749(5.4.2.7)	Yes	No	rn00030: Pentose phosphate pathway///rn01100: Metabolic pathways
R01819(5.3.1.8)	Yes	No	rn00051: Fructose and mannose metabolism///rn00520: Amino sugar and nucleotide sugar metabolism///rn01100: Metabolic pathways///rn01110: Biosynthesis of secondary metabolites
R02568(4.1.2.13)	Yes	No	rn00051: Fructose and mannose metabolism///rn01100: Metabolic pathways///rn01120: Microbial metabolism in diverse environments
R09030(5.3.1.6)	Yes	No	rn00051: Fructose and mannose metabolism///rn01100: Metabolic pathways///rn01120: Microbial metabolism in diverse environments
R00291(5.1.3.2)	Yes	No	rn00052: Galactose metabolism///rn00520: Amino sugar and nucleotide sugar metabolism///rn01100: Metabolic pathways

R00505(5.4.99.9)	Yes	No	rn00052: Galactose metabolism///rn00520: Amino sugar and nucleotide sugar metabolism///rn01100: Metabolic pathways
R00127(2.7.4.3)	Yes	Yes	rn00230: Purine metabolism///rn01100: Metabolic pathways///rn01110: Biosynthesis of secondary metabolites
R03815(1.8.1.4)	Yes	No	rn00260: Glycine, serine and threonine metabolism
R00192(3.3.1.1)	Yes	No	rn00270: Cysteine and methionine metabolism///rn01100: Metabolic pathways
R00177(2.5.1.6)	Yes	No	rn00270: Cysteine and methionine metabolism///rn01100: Metabolic pathways///rn01110: Biosynthesis of secondary metabolites///rn01230: Biosynthesis of amino acids
R08364(1.17.4.1)	Yes	No	rn00480: Glutathione metabolism
R00771(5.3.1.9)	Yes	No	rn00500: Starch and sucrose metabolism///rn01100: Metabolic pathways///rn01110: Biosynthesis of secondary metabolites
R02087(5.1.3.9)	Yes	No	rn00520: Amino sugar and nucleotide sugar metabolism///rn01100: Metabolic pathways
R02239(3.1.3.4)	Yes	No	rn00561: Glycerolipid metabolism///rn00564: Glycerophospholipid metabolism///rn01100: Metabolic pathways///rn01110: Biosynthesis of secondary metabolites
R02055(4.1.1.65)	No	Yes	rn00564: Glycerophospholipid metabolism///rn01100: Metabolic pathways///rn01110: Biosynthesis of secondary metabolites
R00842(1.1.1.94)	No	Yes	rn00564: Glycerophospholipid metabolism///rn01110: Biosynthesis of secondary metabolites
R06522(3.1.3.4)	Yes	No	rn00600: Sphingolipid metabolism
R06520(3.1.3.4)	Yes	No	rn00600: Sphingolipid metabolism///rn01100: Metabolic pathways
R06521(3.1.3.4)	Yes	No	rn00600: Sphingolipid metabolism///rn01100: Metabolic pathways
R00939(1.5.1.3)	No	Yes	rn00670: One carbon pool by folate///rn00790: Folate biosynthesis///rn01100: Metabolic pathways
R01068(4.1.2.13)	Yes	No	rn00680: Methane metabolism///rn00710: Carbon fixation in photosynthetic organisms///rn01100: Metabolic pathways///rn01120: Microbial metabolism in diverse environments///rn01200: Carbon metabolism
R01829(4.1.2.13)	Yes	Yes	rn00710: Carbon fixation in photosynthetic organisms///rn01100: Metabolic pathways///rn01120: Microbial metabolism in diverse environments///rn01200: Carbon metabolism
R06590(2.2.1.1)	Yes	No	rn01051: Biosynthesis of ansamycins///rn01110: Biosynthesis of secondary metabolites
R03596(1.8.1.9)	Yes	No	thioredoxin reductase (NADPH) [EC:1.8.1.9]///thioredoxin reductase (NADPH) [EC:1.8.1.9]
R09372(1.8.1.9)	Yes	No	thioredoxin reductase (NADPH) [EC:1.8.1.9]///thioredoxin reductase (NADPH) [EC:1.8.1.9]
R01567(2.7.1.21)	Yes	No	thymidine kinase [EC:2.7.1.21]
R02099(2.7.1.21)	Yes	No	thymidine kinase [EC:2.7.1.21]
R08233(2.7.1.21)	Yes	No	thymidine kinase [EC:2.7.1.21]
R02101(2.1.1.45)	No	Yes	thymidylate synthase [EC:2.1.1.45]///dihydrofolate reductase / thymidylate synthase [EC:1.5.1.3 2.1.1.45]
R01827(2.2.1.2)	Yes	No	transaldolase [EC:2.2.1.2]///transaldolase / glucose-6-phosphate isomerase [EC:2.2.1.2 5.3.1.9]

R01830(2.2.1.1)	Yes	Yes	transketolase [EC:2.2.1.1]
R01067(2.2.1.1)	Yes	No	transketolase [EC:2.2.1.1]
R00966(2.4.2.9)	Yes	Yes	uracil phosphoribosyltransferase [EC:2.4.2.9]///pyrimidine operon attenuation protein / uracil phosphoribosyltransferase [EC:2.4.2.9]
R00289(2.7.7.9)	Yes	No	UTP--glucose-1-phosphate uridylyltransferase [EC:2.7.7.9]///UDP-sugar pyrophosphorylase [EC:2.7.7.64]///UTP---glucose-1-phosphate uridylyltransferase [EC:2.7.7.9]
R00156(2.7.4.6)	No	Yes	rn00240: Pyrimidine metabolism///rn01100: Metabolic pathways
R00570(2.7.4.6)	No	Yes	rn00240: Pyrimidine metabolism///rn01100: Metabolic pathways
R02326(2.7.4.6)	No	Yes	rn00240: Pyrimidine metabolism///rn01100: Metabolic pathways
R02331(2.7.4.6)	No	Yes	rn00240: Pyrimidine metabolism///rn01100: Metabolic pathways
R02093(2.7.4.6)	No	Yes	rn00240: Pyrimidine metabolism///rn01100: Metabolic pathways
R02738(2.7.1.199)	Yes	Yes	rn00010: Glycolysis / Gluconeogenesis///rn00520: Amino sugar and nucleotide sugar metabolism///rn01100: Metabolic pathways
R01843(2.7.1.11)	Yes	Yes	
R02324	No	Yes	rn00760: Nicotinate and nicotinamide metabolism///rn01100: Metabolic pathways
R00137	No	Yes	rn00760: Nicotinate and nicotinamide metabolism///rn01100: Metabolic pathways
R00549	No	Yes	rn00740: Riboflavin metabolism///rn01100: Metabolic pathways///rn01110: Biosynthesis of secondary metabolites
R00161	No	Yes	rn00740: Riboflavin metabolism///rn01100: Metabolic pathways///rn01110: Biosynthesis of secondary metabolites
R00619	No	Yes	rn00730: Thiamine metabolism///rn01100: Metabolic pathways
R00177	No	Yes	rn00270: Cysteine and methionine metabolism///rn01100: Metabolic pathways///rn01110: Biosynthesis of secondary metabolites///rn01230: Biosynthesis of amino acids
R00173	No	Yes	rn00750: Vitamin B6 metabolism///rn01100: Metabolic pathways
R02971	No	Yes	rn00770: Pantothenate and CoA biosynthesis
R03035	No	Yes	rn00770: Pantothenate and CoA biosynthesis///rn01100: Metabolic pathways
R00130	No	Yes	rn00770: Pantothenate and CoA biosynthesis///rn01100: Metabolic pathways
R03018	No	Yes	rn00770: Pantothenate and CoA biosynthesis///rn01100: Metabolic pathways
R04391	No	Yes	rn00770: Pantothenate and CoA biosynthesis
R04231	No	Yes	rn00770: Pantothenate and CoA biosynthesis///rn01100: Metabolic pathways

Table S4. Names of the enzymes and definition of each reaction involved in the comparison of the MBBs of the three networks under study.

Reaction	Enzyme name	Definition
R00156	ATP:UDP phosphotransferase	ATP + UDP ↔ ADP + UTP
R00158	ATP:UMP phosphotransferase	ATP + UMP ↔ ADP + UDP
R00332	ATP:GMP phosphotransferase	ATP + GMP ↔ ADP + GDP
R00430	GTP:pyruvate 2-O-phosphotransferase	GTP + Pyruvate ↔ GDP + Phosphoenolpyruvate
R00658	2-phospho-D-glycerate hydro-lyase (phosphoenolpyruvate-forming)	2-Phospho-D-glycerate ↔ Phosphoenolpyruvate + H2O
R00842	sn-Glycerol-3-phosphate:NAD+ 2-oxidoreductase	sn-Glycerol 3-phosphate + NAD+ ↔ Glycerone phosphate + NADH + H+
R00844	sn-Glycerol-3-phosphate:NADP+ 2-oxidoreductase	sn-Glycerol 3-phosphate + NADP+ ↔ Glycerone phosphate + NADPH + H+
R00966	UMP:diphosphate phospho-alpha-D-ribosyltransferase	UMP + Diphosphate ↔ Uracil + 5-Phospho-alpha-D-ribose 1-diphosphate
R01049	ATP:D-ribose-5-phosphate diphosphotransferase	ATP + D-Ribose 5-phosphate ↔ AMP + 5-Phospho-alpha-D-ribose 1-diphosphate
R01056	D-ribose-5-phosphate aldose-ketose-isomerase	D-Ribose 5-phosphate ↔ D-Ribulose 5-phosphate
R01061	D-glyceraldehyde-3-phosphate:NAD+ oxidoreductase (phosphorylating)	D-Glyceraldehyde 3-phosphate + Orthophosphate + NAD+ ↔ 3-Phospho-D-glyceroyl phosphate + NADH + H+
R01070	beta-D-fructose-1,6-bisphosphate D-glyceraldehyde-3-phosphate-lyase (glycerone-phosphate-forming)	beta-D-Fructose 1,6-bisphosphate ↔ Glycerone phosphate + D-Glyceraldehyde 3-phosphate
R01229	GMP:diphosphate 5-phospho-alpha-D-ribosyltransferase	GMP + Diphosphate ↔ Guanine + 5-Phospho-alpha-D-ribose 1-diphosphate
R01512	ATP:3-phospho-D-glycerate 1-phosphotransferase	ATP + 3-Phospho-D-glycerate ↔ ADP + 3-Phospho-D-glyceroyl phosphate
R01518	D-phosphoglycerate 2,3-phosphomutase	2-Phospho-D-glycerate ↔ 3-Phospho-D-glycerate
R01529	D-Ribulose-5-phosphate 3-epimerase	D-Ribulose 5-phosphate ↔ D-Xylulose 5-phosphate
R01641	sedoheptulose-7-phosphate:D-glyceraldehyde-3-phosphate glycolaldehyde transferase	Sedoheptulose 7-phosphate + D-Glyceraldehyde 3-phosphate ↔ D-Ribose 5-phosphate + D-Xylulose 5-phosphate
R01829	sedoheptulose 1,7-bisphosphate D-glyceraldehyde-3-phosphate-lyase	Sedoheptulose 1,7-bisphosphate ↔ Glycerone phosphate + D-Erythrose 4-phosphate
R01830	beta-D-Fructose 6-phosphate:D-glyceraldehyde-3-phosphate glycolaldehyde transferase	beta-D-Fructose 6-phosphate + D-Glyceraldehyde 3-phosphate ↔ D-Erythrose 4-phosphate + D-Xylulose 5-phosphate
R01843	ATP:Sedoheptulose 7-phosphate 1-phosphotransferase	ATP + Sedoheptulose 7-phosphate ↔ ADP + Sedoheptulose 1,7-bisphosphate
R02740	alpha-D-Glucose 6-phosphate ketol-isomerase	alpha-D-Glucose 6-phosphate ↔ beta-D-Fructose 6-phosphate

R04779	ATP:D-fructose-6-phosphate 1-phosphotransferase	ATP + beta-D-Fructose 6-phosphate ↔ ADP + beta-D-Fructose 1,6-bisphosphate
R02098	ATP:dUMP phosphotransferase	ATP + dUMP ↔ ADP + dUDP
R02331	ATP:dUDP phosphotransferase	ATP + dUDP ↔ ADP + dUTP
R00512	ATP:CMP phosphotransferase	ATP + CMP ↔ ADP + CDP
R00570	ATP:CDP phosphotransferase	ATP + CDP ↔ ADP + CTP
R02019	2'-Deoxyguanosine 5'-diphosphate:oxidized-thioredoxin 2'-oxidoreductase	dGDP + Thioredoxin disulfide + H2O ↔ GDP + Thioredoxin
R00127	ATP:AMP phosphotransferase	ATP + AMP ↔ 2 ADP
R00190	AMP:diphosphate phospho-D-ribosyltransferase	AMP + Diphosphate ↔ Adenine + 5-Phospho-alpha-D-ribose 1-diphosphate
R01138	dATP:pyruvate 2-O-phosphotransferase	dATP + Pyruvate ↔ dADP + Phosphoenolpyruvate
R02017	2'-Deoxyadenosine 5'-diphosphate:oxidized-thioredoxin 2'-oxidoreductase	dADP + Thioredoxin disulfide + H2O ↔ Thioredoxin + ADP
R00936	5,6,7,8-tetrahydrofolate:NAD+ oxidoreductase	Tetrahydrofolate + NAD+ ↔ Dihydrofolate + NADH + H+
R00939	5,6,7,8-tetrahydrofolate:NADP+ oxidoreductase	Tetrahydrofolate + NADP+ ↔ Dihydrofolate + NADPH + H+
R00945	5,10-Methylenetetrahydrofolate:glycine hydroxymethyltransferase	5,10-Methylenetetrahydrofolate + Glycine + H2O ↔ Tetrahydrofolate + L-Serine
R02101	5,10-Methylenetetrahydrofolate:dUMP C-methyltransferase	dUMP + 5,10-Methylenetetrahydrofolate ↔ Dihydrofolate + dTMP
R02235	dihydrofolate:NAD+ oxidoreductase	Dihydrofolate + NAD+ ↔ Folate + NADH + H+
R02236	dihydrofolate:NADP+ oxidoreductase	Dihydrofolate + NADP+ ↔ Folate + NADPH + H+
R00137	ATP:nicotinamide-nucleotide adenylyltransferase	ATP + Nicotinamide D-ribonucleotide ↔ Diphosphate + NAD+
R02093	ATP:dTDP phosphotransferase	ATP + dTDP ↔ ADP + dTTP
R02094	ATP:dTMP phosphotransferase	ATP + dTMP ↔ ADP + dTDP
R00014	pyruvate:thiamin diphosphate acetaldehydetransferase (decarboxylating)	Pyruvate + Thiamin diphosphate ↔ 2-(alpha-Hydroxyethyl)thiamine diphosphate + CO2
R00230	acetyl-CoA:phosphate acetyltransferase	Acetyl-CoA + Orthophosphate ↔ CoA + Acetyl phosphate
R00315	ATP:acetate phosphotransferase	ATP + Acetate ↔ ADP + Acetyl phosphate
R02569	acetyl-CoA:enzyme N6-(dihydrolipoyl)lysine S-acetyltransferase	Acetyl-CoA + Enzyme N6-(dihydrolipoyl)lysine ↔ CoA + [Dihydrolipoyllysine-residue acetyltransferase] S-acetyldihydrolipoyllysine
R03270	pyruvate dehydrogenase	2-(alpha-Hydroxyethyl)thiamine diphosphate + Enzyme N6-(lipoyl)lysine ↔ [Dihydrolipoyllysine-residue acetyltransferase] S-acetyldihydrolipoyllysine + Thiamin diphosphate

R07618	enzyme N6-(dihydrolipoyl)lysine:NAD+ oxidoreductase	Enzyme N6-(dihydrolipoyl)lysine + NAD+ ↔ Enzyme N6-(lipoyl)lysine + NADH + H+
R01126	inosine 5'-monophosphate phosphohydrolase	IMP + H2O ↔ Inosine + Orthophosphate
R01132	IMP:diphosphate phospho-D-ribosyltransferase	IMP + Diphosphate ↔ Hypoxanthine + 5-Phospho-alpha-D-ribose 1-diphosphate
R01863	inosine:phosphate alpha-D-ribosyltransferase	Inosine + Orthophosphate ↔ Hypoxanthine + alpha-D-Ribose 1-phosphate
R02142	XMP:pyrophosphate phosphoribosyltransferase	Xanthosine 5'-phosphate + Diphosphate ↔ Xanthine + 5-Phospho-alpha-D-ribose 1-diphosphate
R02297	Xanthosine:orthophosphate ribosyltransferase	Xanthosine + Orthophosphate ↔ Xanthine + alpha-D-Ribose 1-phosphate
R02719	xanthosine 5'-phosphate phosphohydrolase	Xanthosine 5'-phosphate + H2O ↔ Xanthosine + Orthophosphate
R00921	propanoyl-CoA:phosphate propanoyltransferase	Propanoyl-CoA + Orthophosphate ↔ Propanoyl phosphate + CoA
R01353	ATP:propanoate phosphotransferase	ATP + Propanoate ↔ ADP + Propanoyl phosphate
R00512	ATP:CMP phosphotransferase	ATP + CMP ↔ ADP + CDP
R00158	ATP:UMP phosphotransferase	ATP + UMP ↔ ADP + UDP
R00289	UTP:alpha-D-glucose-1-phosphate uridylyltransferase	UTP + D-Glucose 1-phosphate ↔ Diphosphate + UDP-glucose
R00291	UDP-glucose 4-epimerase;///UDP-alpha-D-glucose 4-epimerase	UDP-glucose ↔ UDP-alpha-D-galactose
R00332	ATP:GMP phosphotransferase	ATP + GMP ↔ ADP + GDP
R00430	GTP:pyruvate 2-O-phosphotransferase	GTP + Pyruvate ↔ GDP + Phosphoenolpyruvate
R00505	UDP-alpha-D-galactopyranose furanomutase	UDP-alpha-D-galactose ↔ UDP-alpha-D-galactofuranose
R00658	2-phospho-D-glycerate hydro-lyase (phosphoenolpyruvate-forming)	2-Phospho-D-glycerate ↔ Phosphoenolpyruvate + H2O
R00959	alpha-D-Glucose 1-phosphate 1,6-phosphomutase	D-Glucose 1-phosphate ↔ alpha-D-Glucose 6-phosphate
R00966	UMP:diphosphate phospho-alpha-D-ribosyltransferase	UMP + Diphosphate ↔ Uracil + 5-Phospho-alpha-D-ribose 1-diphosphate
R01015	D-glyceraldehyde-3-phosphate aldose-ketose-isomerase	D-Glyceraldehyde 3-phosphate ↔ Glycerone phosphate
R01049	ATP:D-ribose-5-phosphate diphosphotransferase	ATP + D-Ribose 5-phosphate ↔ AMP + 5-Phospho-alpha-D-ribose 1-diphosphate
R01056	D-ribose-5-phosphate aldose-ketose-isomerase	D-Ribose 5-phosphate ↔ D-Ribulose 5-phosphate
R01057	D-Ribose 1,5-phosphomutase	alpha-D-Ribose 1-phosphate ↔ D-Ribose 5-phosphate
R01058	D-glyceraldehyde 3-phosphate:NADP+ oxidoreductase	D-Glyceraldehyde 3-phosphate + NADP+ + H2O ↔ 3-Phospho-D-glycerate + NADPH + H+
R01061	D-glyceraldehyde-3-phosphate:NAD+ oxidoreductase (phosphorylating)	D-Glyceraldehyde 3-phosphate + Orthophosphate + NAD+ ↔ 3-Phospho-D-glyceroyl phosphate + NADH + H+

R01066	2-deoxy-D-ribose-5-phosphate acetaldehyde-lyase (D-glyceraldehyde-3-phosphate-forming)	2-Deoxy-D-ribose 5-phosphate ↔ D-Glyceraldehyde 3-phosphate + Acetaldehyde
R01067	D-Fructose 6-phosphate:D-glyceraldehyde-3-phosphate glycolaldehyde transferase	D-Fructose 6-phosphate + D-Glyceraldehyde 3-phosphate ↔ D-Erythrose 4-phosphate + D-Xylulose 5-phosphate
R01068	D-fructose-1,6-bisphosphate D-glyceraldehyde-3-phosphate-lyase (glycerone-phosphate-forming)	D-Fructose 1,6-bisphosphate ↔ Glycerone phosphate + D-Glyceraldehyde 3-phosphate
R01070	beta-D-fructose-1,6-bisphosphate D-glyceraldehyde-3-phosphate-lyase (glycerone-phosphate-forming)	beta-D-Fructose 1,6-bisphosphate ↔ Glycerone phosphate + D-Glyceraldehyde 3-phosphate
R01227	guanosine 5'-monophosphate phosphohydrolase	GMP + H2O ↔ Guanosine + Orthophosphate
R01229	GMP:diphosphate 5-phospho-alpha-D-ribosyltransferase	GMP + Diphosphate ↔ Guanine + 5-Phospho-alpha-D-ribose 1-diphosphate
R01229	GMP:diphosphate 5-phospho-alpha-D-ribosyltransferase	GMP + Diphosphate ↔ Guanine + 5-Phospho-alpha-D-ribose 1-diphosphate
R01512	ATP:3-phospho-D-glycerate 1-phosphotransferase	ATP + 3-Phospho-D-glycerate ↔ ADP + 3-Phospho-D-glyceroyl phosphate
R01518	D-phosphoglycerate 2,3-phosphomutase	2-Phospho-D-glycerate ↔ 3-Phospho-D-glycerate
R01529	D-Ribulose-5-phosphate 3-epimerase	D-Ribulose 5-phosphate ↔ D-Xylulose 5-phosphate
R01641	sedoheptulose-7-phosphate:D-glyceraldehyde-3-phosphate glycolaldehyde transferase	Sedoheptulose 7-phosphate + D-Glyceraldehyde 3-phosphate ↔ D-Ribose 5-phosphate + D-Xylulose 5-phosphate
R01819	D-mannose-6-phosphate aldose-ketose-isomerase	D-Mannose 6-phosphate ↔ beta-D-Fructose 6-phosphate
R01827	sedoheptulose-7-phosphate:D-glyceraldehyde-3-phosphate glyceronetransferase	Sedoheptulose 7-phosphate + D-Glyceraldehyde 3-phosphate ↔ D-Erythrose 4-phosphate + beta-D-Fructose 6-phosphate
R01829	sedoheptulose 1,7-bisphosphate D-glyceraldehyde-3-phosphate-lyase	Sedoheptulose 1,7-bisphosphate ↔ Glycerone phosphate + D-Erythrose 4-phosphate
R01830	beta-D-Fructose 6-phosphate:D-glyceraldehyde-3-phosphate glycolaldehyde transferase	beta-D-Fructose 6-phosphate + D-Glyceraldehyde 3-phosphate ↔ D-Erythrose 4-phosphate + D-Xylulose 5-phosphate
R01843	ATP:Sedoheptulose 7-phosphate 1-phosphotransferase	ATP + Sedoheptulose 7-phosphate ↔ ADP + Sedoheptulose 1,7-bisphosphate
R01967	ATP:deoxyguanosine 5'-phosphotransferase	ATP + Deoxyguanosine ↔ ADP + dGMP
R01968	2'-deoxyguanosine 5'-monophosphate phosphohydrolase	dGMP + H2O ↔ Deoxyguanosine + Orthophosphate
R01969	Deoxyguanosine:orthophosphate ribosyltransferase	Deoxyguanosine + Orthophosphate ↔ Guanine + 2-Deoxy-D-ribose 1-phosphate
R02018	2'-Deoxyuridine 5'-diphosphate:oxidized-thioredoxin 2'-oxidoreductase	dUDP + Thioredoxin disulfide + H2O ↔ Thioredoxin + UDP
R02019	2'-Deoxyguanosine 5'-diphosphate:oxidized-thioredoxin 2'-oxidoreductase	dGDP + Thioredoxin disulfide + H2O ↔ GDP + Thioredoxin
R02090	ATP:dGMP phosphotransferase	ATP + dGMP ↔ ADP + dGDP
R02098	ATP:dUMP phosphotransferase	ATP + dUMP ↔ ADP + dUDP

R02099	ATP:deoxyuridine 5'-phosphotransferase	ATP + Deoxyuridine ↔ ADP + dUMP
R02102	2'-deoxyuridine 5'-monophosphate phosphohydrolase	dUMP + H2O ↔ Deoxyuridine + Orthophosphate
R02102	2'-deoxyuridine 5'-monophosphate phosphohydrolase	dUMP + H2O ↔ Deoxyuridine + Orthophosphate
R02147	guanosine:phosphate alpha-D-ribosyltransferase	Guanosine + Orthophosphate ↔ Guanine + alpha-D-Ribose 1-phosphate
R02484	deoxyuridine:orthophosphate 2-deoxy-D-ribosyltransferase;///deoxyuridine:orthophosphate ribosyltransferase	Deoxyuridine + Orthophosphate ↔ Uracil + 2-Deoxy-D-ribose 1-phosphate
R02568	D-fructose 1-phosphate D-glyceraldehyde-3-phosphate-lyase	D-Fructose 1-phosphate ↔ Glycerone phosphate + D-Glyceraldehyde
R02739	alpha-D-Glucose 6-phosphate ketol-isomerase	alpha-D-Glucose 6-phosphate ↔ beta-D-Glucose 6-phosphate
R02740	alpha-D-Glucose 6-phosphate ketol-isomerase	alpha-D-Glucose 6-phosphate ↔ beta-D-Fructose 6-phosphate
R02749	2-deoxy-D-ribose 1-phosphate 1,5-phosphomutase	2-Deoxy-D-ribose 1-phosphate ↔ 2-Deoxy-D-ribose 5-phosphate
R03321	beta-D-Glucose 6-phosphate ketol-isomerase	beta-D-Glucose 6-phosphate ↔ beta-D-Fructose 6-phosphate
R04779	ATP:D-fructose-6-phosphate 1-phosphotransferase	ATP + beta-D-Fructose 6-phosphate ↔ ADP + beta-D-Fructose 1,6-bisphosphate
R01664	2'-deoxycytidine 5'-monophosphate phosphohydrolase	dCMP + H2O ↔ Deoxycytidine + Orthophosphate
R01664	2'-deoxycytidine 5'-monophosphate phosphohydrolase	dCMP + H2O ↔ Deoxycytidine + Orthophosphate
R01665	ATP:dCMP phosphotransferase	ATP + dCMP ↔ ADP + dCDP
R01666	ATP:deoxycitidine 5'-phosphotransferase	ATP + Deoxycytidine ↔ ADP + dCMP
R01667	dCDP nucleotidohydrolase	dCDP + H2O ↔ dCMP + Orthophosphate
R00127	ATP:AMP phosphotransferase	ATP + AMP ↔ 2 ADP
R00183	adenosine 5'-monophosphate phosphohydrolase	AMP + H2O ↔ Adenosine + Orthophosphate
R00185	ATP:adenosine 5'-phosphotransferase	ATP + Adenosine ↔ ADP + AMP
R00190	AMP:diphosphate phospho-D-ribosyltransferase	AMP + Diphosphate ↔ Adenine + 5-Phospho-alpha-D-ribose 1-diphosphate
R01138	dATP:pyruvate 2-O-phosphotransferase	dATP + Pyruvate ↔ dADP + Phosphoenolpyruvate
R01547	ATP:dAMP phosphotransferase	ATP + dAMP ↔ ADP + dADP
R01547	ATP:dAMP phosphotransferase	ATP + dAMP ↔ ADP + dADP
R01561	adenosine:phosphate alpha-D-ribosyltransferase	Adenosine + Orthophosphate ↔ Adenine + alpha-D-Ribose 1-phosphate
R02017	2'-Deoxyadenosine 5'-diphosphate:oxidized-thioredoxin 2'-oxidoreductase	dADP + Thioredoxin disulfide + H2O ↔ Thioredoxin + ADP
R02088	2'-deoxyadenosine 5'-monophosphate phosphohydrolase	dAMP + H2O ↔ Deoxyadenosine + Orthophosphate

R02089	ATP:deoxyadenosine 5'-phosphotransferase	ATP + Deoxyadenosine ↔ ADP + dAMP
R02557	Deoxyadenosine:orthophosphate ribosyltransferase	Deoxyadenosine + Orthophosphate ↔ Adenine + 2-Deoxy-D-ribose 1-phosphate
R00942	Tetrahydrofolate:L-glutamate gamma-ligase (ADP-forming)	ATP + Tetrahydrofolate + L-Glutamate ↔ ADP + Orthophosphate + THF-L-glutamate
R00945	5,10-Methylenetetrahydrofolate:glycine hydroxymethyltransferase	5,10-Methylenetetrahydrofolate + Glycine + H2O ↔ Tetrahydrofolate + L-Serine
R01220	5,10-methylenetetrahydrofolate:NADP+ oxidoreductase	5,10-Methylenetetrahydrofolate + NADP+ ↔ 5,10-Methenyltetrahydrofolate + NADPH
R01655	5,10-Methenyltetrahydrofolate 5-hydrolase (decyclizing)	5,10-Methenyltetrahydrofolate + H2O ↔ 10-Formyltetrahydrofolate + H+
R03940	10-Formyltetrahydrofolate:L-methionyl-tRNA N-formyltransferase	L-Methionyl-tRNA + 10-Formyltetrahydrofolate ↔ Tetrahydrofolate + N-Formylmethionyl-tRNA
R04241	tetrahydropteroyl-gamma-polyglutamate:L-glutamate gamma-ligase (ADP-forming)	ATP + THF-polyglutamate(n) + L-Glutamate ↔ ADP + Orthophosphate + THF-polyglutamate(n+1)
R00137	ATP:nicotinamide-nucleotide adenylyltransferase	ATP + Nicotinamide D-ribonucleotide ↔ Diphosphate + NAD+
R01271	nicotinamide-D-ribonucleotide:diphosphate phospho-alpha-D-ribosyltransferase	Nicotinamide D-ribonucleotide + Diphosphate ↔ Nicotinamide + 5-Phospho-alpha-D-ribose 1-diphosphate
R02294	N-Ribosylnicotinamide:orthophosphate ribosyltransferase	Nicotinamide-beta-riboside + Orthophosphate ↔ Nicotinamide + alpha-D-Ribose 1-phosphate
R02323	nicotinamide ribonucleotide phosphohydrolase	Nicotinamide D-ribonucleotide + H2O ↔ Nicotinamide-beta-riboside + Orthophosphate
R01567	ATP:thymidine 5'-phosphotransferase	ATP + Thymidine ↔ ADP + dTMP
R01569	thymidylate 5'-phosphohydrolase	dTMP + H2O ↔ Thymidine + Orthophosphate
R02094	ATP:dTMP phosphotransferase	ATP + dTMP ↔ ADP + dTDP
R00435	ATP:polynucleotide adenylyltransferase;///ATP:RNA adenylyltransferase	ATP + RNA ↔ Diphosphate + RNA
R00441	GTP:RNA guanylyltransferase (DNA-directed);///GTP:RNA guanylyltransferase (RNA-directed)	GTP + RNA ↔ Diphosphate + RNA
R00375	Deoxyadenosine 5'-triphosphate:DNA deoxynucleotidyltransferase (DNA-directed)	dATP + DNA ↔ Diphosphate + DNA
R00376	Deoxyguanosine 5'-triphosphate:DNA deoxynucleotidyltransferase (DNA-directed)	dGTP + DNA ↔ Diphosphate + DNA

Figure S1. Full size representation of the reaction graph of the proposed theoretical minimal metabolic network represented in Figure 1

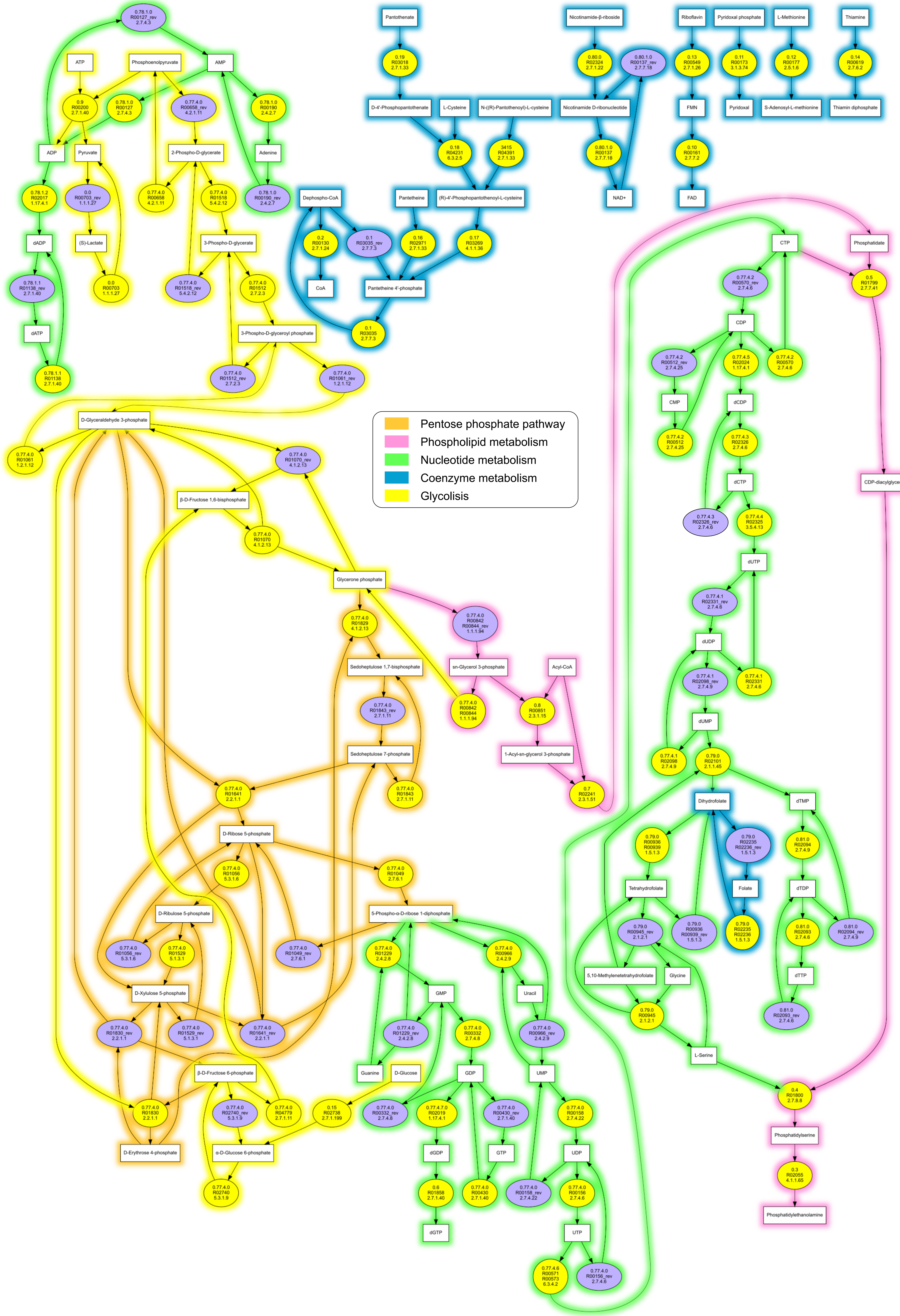

Figure S2. The m-DAG of “Ca. *Nasuia deltocephalinicola”*.

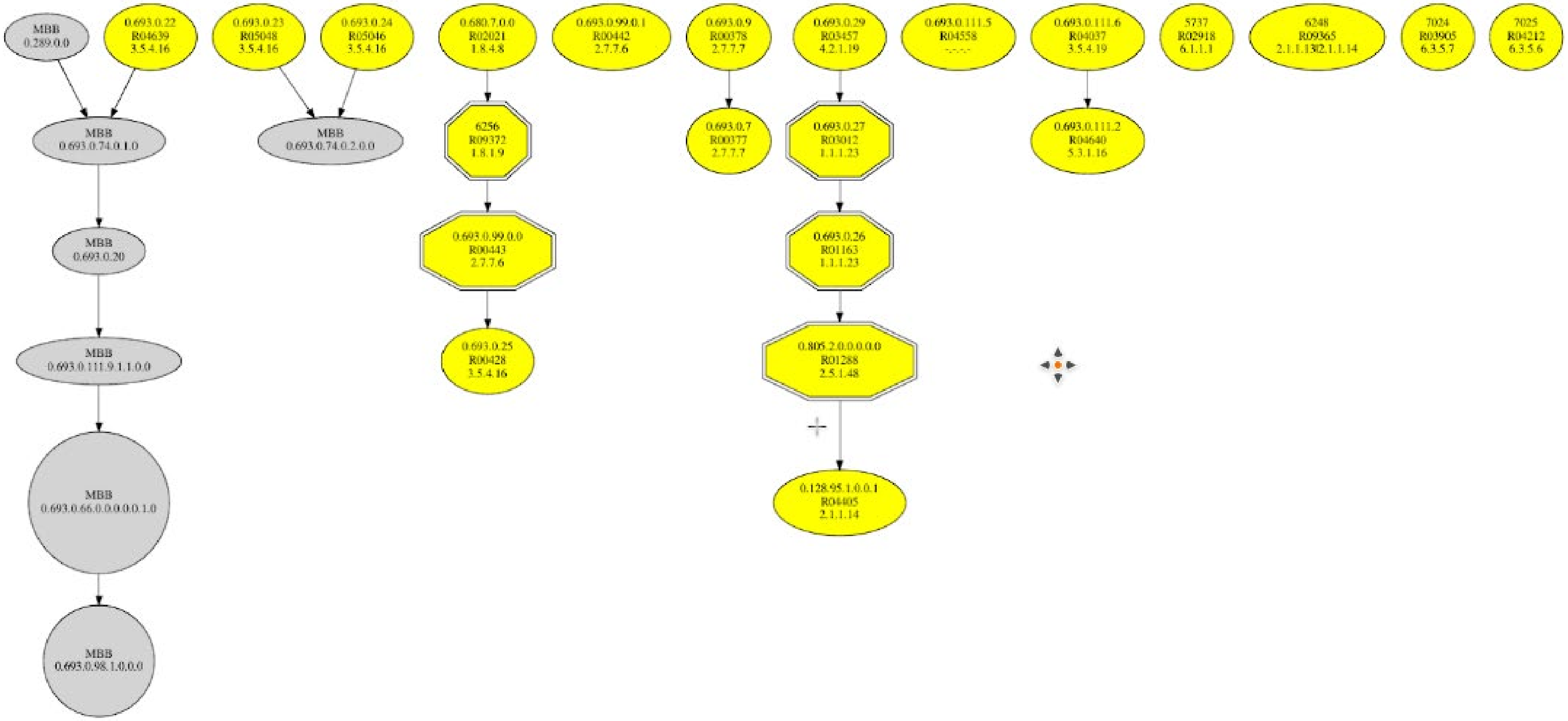

Supplement: Supplementary file 1 [file biology-10-00005-s001.pdf]
